# Time to Intervene: A Continuous-Time Approach to Network Analysis and Centrality

**DOI:** 10.1007/s11336-021-09767-0

**Published:** 2021-06-24

**Authors:** Oisín Ryan, Ellen L. Hamaker

**Affiliations:** Utrecht University, Padualaan 14, 3584 CH, Utrecht, The Netherlands

**Keywords:** dynamical network analysis, continuous-time modeling, centrality, intensive longitudinal data, experience sampling methodology

## Abstract

Network analysis of ESM data has become popular in clinical psychology. In this approach, discrete-time (DT) vector auto-regressive (VAR) models define the network structure with centrality measures used to identify intervention targets. However, VAR models suffer from time-interval dependency. Continuous-time (CT) models have been suggested as an alternative but require a conceptual shift, implying that DT-VAR parameters reflect total rather than direct effects. In this paper, we propose and illustrate a CT network approach using CT-VAR models. We define a new network representation and develop centrality measures which inform intervention targeting. This methodology is illustrated with an ESM dataset.

**Supplementary Information:**

The online version contains supplementary material available at 10.1007/s11336-021-09767-0.

Dynamical networks are a popular approach for the analysis of experience sampling data in psychology (Bringmann et al., 2013; Borsboom & Cramer, 2013). In this approach, researchers typically make use of the discrete-time (DT) first-order vector auto-regressive (VAR) model, with the estimated lagged parameters of this model treated as edges directly connecting nodes in the network. In clinical psychology in particular, dynamical network analyses have been promoted as an aid in developing personalized treatments for psychopathology. To facilitate this, *centrality measures* based on parameter estimates are used to identify which variable in the network represents the most promising target for future interventions (Bringmann et al., 2013; Fisher & Boswell, 2016; Kroeze et al., 2017; Epskamp et al., 2018; Rubel et al., 2018; Bak et al., 2016; Bringmann et al., 2015; Bastiaansen et al., 2019; Fisher et al., 2017; Christian et al., 2019).

However, it is well known that the DT-VAR model suffers from the problem of *time-interval dependency* (Gollob & Reichardt, 1987), which entails that the estimated lagged parameters are a function of the amount of time that elapses between repeated measurements. This problem can be resolved by modeling psychological processes as unfolding *continuously* over time using continuous-time (CT) models that explicitly account for the time-interval dependency of lagged parameters (e.g., Boker, 2002; Oud & Delsing, 2010; van Montfort et al., 2018; Ryan et al., 2018; Voelkle et al., 2012). Such models can easily deal with unequal intervals, and can be used to derive how lagged parameters are expected to evolve over a whole range of time-interval values. Yet, taking a CT perspective also entails a conceptual shift, in that lagged regression parameters at any interval should be interpreted as *total* rather than *direct* relationships (Aalen et al., 2012; Aalen et al., 2016; Deboeck & Preacher, 2016). While the general consequences of this have been discussed elsewhere, the consequences for the network approach have yet to be elucidated. This leaves a number of open questions, most notably: what are the implications of the CT perspective for current centrality measures? How can CT models be used to yield novel insights into a dynamical network? And how can we use CT models to choose intervention targets?

The aim of the current paper is to answer these questions. This paper is organized as follows. In the first section, we provide an overview of the DT-VAR model and how path-specific effects and centrality measures are used to identify intervention targets in practice. Moreover, we discuss the time-interval problem that is associated with this approach. In the second section, we present the CT-VAR model as an alternative approach to dynamical network analysis, and explore the consequences of this. In the third section, we introduce new fit-for-purpose centrality measures that both reflect the CT nature of the underlying process and have a clear and direct conceptual link to interventions and the choice of optimal intervention targets. Finally, we demonstrate the application of CT network analysis using empirical data. For simplicity, the developments in this paper focus on single-subject models, though the critiques and measures developed here generalize in a straightforward way to within-subjects parameters of multilevel models.

## Current Practice: DT-VAR Networks

Researchers who adopt a network perspective on psychological phenomena often use the parameters of (single-subject or multilevel) first-order discrete-time vector auto-regressive (DT-VAR) models to suggest intervention targets (Bringmann et al., 2013; Pe et al., 2015; Fisher & Boswell, 2016; Kroeze et al., 2017; Rubel et al., 2018; Bak et al., 2016). In this section, we describe this practice. We present the model itself and describe two ways in which researchers have used this model to find intervention targets, that is, through considering path-specific effects and through computing centrality measures. We will show how these two practices are connected, as this insight will prove useful later when considering how CT models could be used in an analogous way. Furthermore, we elaborate on the time-interval problem, and discuss how this issue casts doubt on the appropriateness of current practice, which motivates the developments presented in the remainder of this paper.

### The DT-VAR Model

The DT-VAR model is a single-subject time-series model that describes dynamic relationships between variables measured repeatedly over time. Lagged regression parameters encode the effect of a variable on itself (an auto-regressive effect) or another variable (a cross-lagged effect) at the next measurement occasion (i.e., at a lag of one). This model can be written as

1 $$\begin{aligned} {\varvec{Y}}_\tau = {\varvec{c}} + \varvec{\Phi } {\varvec{Y}}_{\tau -1} + \varvec{\epsilon }_\tau \end{aligned}$$

where given *p* variables, the $p \times 1$ vector of random variables ${\varvec{Y}}$ at occasion $\tau$ is regressed on the $p \times 1$ vector of those same variables at the previous occasion, ${\varvec{Y}}_{\tau -1}$. The $p \times p$ matrix of lagged regression parameters is denoted $\varvec{\Phi }$, while the $p \times 1$ vectors ${\varvec{c}}$ and $\varvec{\epsilon }_\tau$ denote the intercepts and random shocks, respectively, the latter being normally distributed with mean zero and variance-covariance matrix $\varvec{\Psi }$ (Hamilton, 1994). The multivariate mean of the DT-VAR model $\varvec{\mu }$ can be expressed as a function of the intercepts and lagged parameters ($\varvec{\mu } = ({\varvec{I}} - \Phi )^{-1}{\varvec{c}}$) and can be thought of as the equilibrium or attractor of the dynamical system. If we assume the data are centered, the intercept term can be omitted (${\varvec{c}} = {\varvec{0}}$), a convention we will adopt throughout the remainder of the paper.

In qualitative terms, the model describes a system where the random shocks $\varvec{\epsilon }_\tau$ push the system away from its equilibrium, and the lagged parameters $\varvec{\Phi }$ determine how the variables react to these shocks, eventually returning them to equilibrium over time (for more details, see, among others, Ryan et al., 2018; Strogatz, 2015; Haslbeck et al., in press). A distinction can be made between DT-VAR models which exhibit “positive” and “negative” auto-regression: in the former case, the system returns to equilibrium in an exponential fashion over time; In the latter, variables switch their sign (from positive to negative and vice versa) at each subsequent occasion. In the univariate case, this is determined by the sign of the auto-regressive parameter $\phi$, but in the multivariate case by the sign of the eigenvalues of $\varvec{\Phi }$. Positive auto-regression systems are found in many psychological time-series applications (e.g., Bringmann et al., 2013; Koval & Kuppens, 2012; Oravecz & Tuerlinckx, 2011). In this paper, we will assume that our system of interest exhibits positive auto-regression. Two crucial assumptions of the DT-VAR model in general are that the same amount of time (denoted $\Delta t$) elapses between two subsequent measurement occasions, and that the underlying process is stationary, which entails that the means, variance and covariances, and lagged regression parameters remain the same over time.^1^

**Fig. 1:**
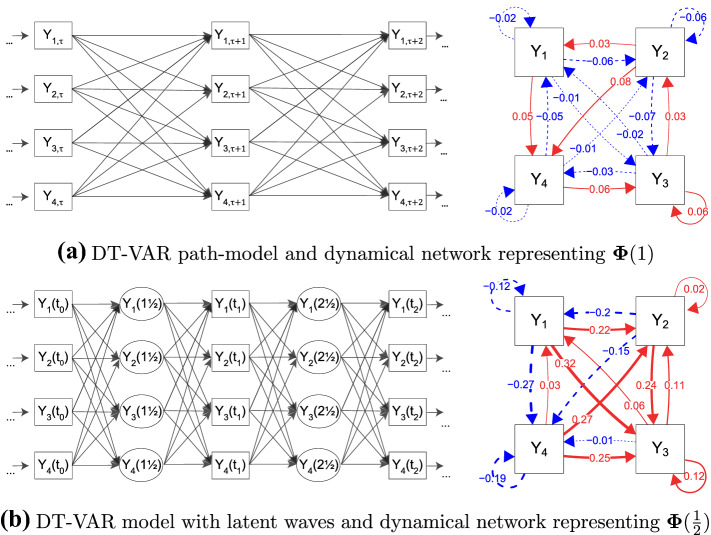
Path model (left-hand side) and network (right-hand side) representations of two four-variable DT-VAR models. In the path models, the presence of an arrow linking two variables denotes some nonzero dependency between them, conditional on all variables at the previous wave. For the networks, the arrows represent auto-regressive and cross-lagged regression parameters in a first-order DT-VAR model. Solid red arrows denote parameters while dashed blue arrows represent negative parameters.

The DT-VAR model can be represented as either a path model, as shown in the left-hand panel of Fig. 1a, or as a dynamical network structure, as shown in the right-hand panel, where the nodes represent the random variables, and the edges represent the values of the lagged parameters $\varvec{\Phi }$ (Bringmann et al., 2013; Epskamp et al., 2018). The lagged parameters in $\varvec{\Phi }$ are typically interpreted as *direct effects* of these variables on each other over time. As an example, take it that the four variables in Fig. 1a represent (repeated measurements of) *Stress* ($Y_1$), *Anxiety* ($Y_2$), *Self-Consciousness* ($Y_3$) and feelings of *Physical Discomfort* ($Y_4$). We will refer throughout to the dynamical system composed of these four time-varying processes as the *Stress-Discomfort* system. We can see from the parameter values in the dynamical network that all variables share reciprocal cross-lagged relationships with all other variables, resulting in a completely connected network. Typically, a cross-lagged parameter such as $\phi _{41} = 0.05$ would be interpreted as the direct effect of current Stress ($Y_{1,\tau }$) on Physical Discomfort at the next measurement occasion ($Y_{4,{\tau + 1}}$), conditional on (i.e., controlling for) current feelings of Anxiety, Self-Consciousness and Physical Discomfort $(Y_{2,\tau }, Y_{3,\tau }, Y_{4,{\tau }})$. This parameter is weakly positive, leading to the interpretation that a high level of current Stress has a small positive direct effect on feelings of Physical Discomfort at the next occasion.

### Intervention Targets from DT-VAR Models

To identify which variables should be considered targets for an intervention based on a DT-VAR model, psychology researchers have mainly used two approaches: (a) *path-specific effects*, which are inspired by the SEM literature (Bollen, 1987); and (b) *centrality measures*, which come from the network analysis literature (Freeman, 1978; Opsahl et al., 2010).

Path-specific effects have been used to describe the *total, direct* and *indirect* effects of one variable on another, and can be calculated using the well-known *path-tracing rules* from the SEM literature (Bollen, 1987). For instance, the total effect of Stress levels now ($Y_{1,\tau }$) on Physical Discomfort two measurement occasions later ($Y_{4,\tau +2}$) is the sum of the direct effect pathways (i.e., $Y_{1,\tau } \rightarrow Y_{4,\tau +1} \rightarrow Y_{4,\tau +2}$, and $Y_{1,\tau } \rightarrow Y_{1,\tau +1} \rightarrow Y_{4,\tau +2}$), and the indirect effect pathways through the mediating variables Anxiety and Self-Consciousness (i.e., $Y_{1,\tau } \rightarrow Y_{2,\tau +1} \rightarrow Y_{4,\tau +2}$, and $Y_{1,\tau } \rightarrow Y_{3,\tau +1} \rightarrow Y_{4,\tau +2}$, respectively; Cole & Maxwell, 2003). If we interpret $\varvec{\Phi }$ parameters as direct causal effects, we may suggest that interventions should target variables that have strong direct or total effects on others. Alternatively, we could search for those mediators through which the strongest indirect effects pass (Groen et al., 2020; Bernat et al., 2007; Bramsen et al., 2013). For instance, based on the parameters in Fig. 1a, we might suggest Anxiety as an intervention target due to the relatively strong *lag-one direct effect* on Physical Discomfort ($\phi _{42} = .08$), or because it is a mediator of the largest *lag-two* indirect effect, from Stress to Physical Discomfort ($Y_{1,\tau } \rightarrow Y_{2,\tau + 1} \rightarrow Y_{4,\tau +2} = -.005$).

An alternative approach to finding a target for intervention comes from the network approach and is based on *centrality measures* (e.g., Bringmann et al., 2013; Fisher & Boswell, 2016; Kroeze et al., 2017; Epskamp et al., 2018; Rubel et al., 2018; Bak et al., 2016; Bringmann et al., 2015; Bastiaansen et al., 2019). Centrality measures are used to summarize the relations a particular variable has with the network as a whole, typically summing over the individual relations that variable has with all other variables in the network. While the precise connection between path-specific effects and centrality measures for DT-VAR models has not yet been described in the literature a close inspection of the computation and interpretation of many popular centrality measures reveals that they are very similar to path-tracing effects: specifically, many centrality measures are interpreted as capturing either *total*, *direct* or *indirect* effects, and in turn, these measures are often closely related to summaries of the corresponding path-specific effects. Here, we will mention three such measures; for the exact connection between these measures and path-tracing effects, the reader is referred to Appendix A.

First, the *Two-Step Expected Influence* measure (${\textit{EI}}^{(2)}_{i}$; Robinaugh et al., 2016; Kaiser & Laireiter, 2018) is typically interpreted as a summary of *total effects* emanating from the variable $Y_i$. In path-tracing terms, it is the sum of lag-one direct effects and lag-two total effects. As such, variables with a high Two-Step Expected Influence could be expected to exert a high overall influence on the system, making it an attractive intervention target. Second, the *One-Step Expected Influence* (${\textit{EI}}^{(1)}_{i}$; Robinaugh et al., 2016; Kaiser & Laireiter, 2018) and *Out-Strength centrality* (Opsahl et al., 2010) measures are interpreted as summarizing direct effects. They are both sums of lag-one direct effects, with the latter taking the absolute value (and so, we will calculate only the expected influence measure in the remainder). Third, *Betweenness Centrality* (BC) is interpreted as indicating the degree to which a variable funnels information flow, similar to how mediating variables funnel *indirect effects* (e.g., Bringmann et al., 2013; Opsahl et al., 2010; Freeman, 1977). This measure is conceptually similar to determining which variables are strong mediators, although paths are calculated by summing, rather than multiplying parameters, as in path-tracing rules. The first column of Table 1 contains the value of these three centrality metrics for each node in the Stress-Discomfort network shown in Fig. 1a.

**Table 1:** Two-Step Expected Influence (${\textit{EI}}^{(2)}$), One-Step Expected Influence (${\textit{EI}}^{(1)}$) and Betweenness Centrality (${\textit{BC}}$) for each of the four variables in the 1-h ($\varvec{\Phi }(\Delta t = 1)$, Fig. 1a) and half-hour ($\varvec{\Phi }(\Delta t = 0.5)$, Fig. 1b) networks.

	$\Delta t = 1$			$\Delta t = 0.5$		
	${\textit{EI}}^{(2)}$	${\textit{EI}}^{(1)}$	${\textit{BC}}$	${\textit{EI}}^{(2)}$	${\textit{EI}}^{(1)}$	${\textit{BC}}$
Stress	0.245	0.274	0	$-$0.025	$-$0.029	**1**
Anxiety	$-$0.071	$-$0.109	**3**	**0**.**035**	**0**.**038**	**1**
Self-Consciousness	0.125	0.152	0	$-$0.024	$-$0.027	0
Physical discomfort	**0**.**555**	**0**.**557**	0	0.008	$-$0.002	**1**

In each column, the largest centrality values are highlighted in bold.

### The Time-Interval Problem and its Consequences

From this review, it is clear that network analysis based on the DT-VAR model relies critically on the interpretation of a cross-lagged regression parameter as *the direct effect* of one process on another over time (for similar interpretations of DT-VAR models in the time series and panel data literature, see Cole & Maxwell, 2003; Hamaker et al., 2015; Bulteel et al., 2016). Of course, the interpretation of any model parameter estimated from observational data as a causal effect or as informative about hypothetical interventions should be approached with due caution. Developments in the causal inference literature have shown that such interpretations are highly dependent on the validity of assumptions regarding, among others, the ignorability of unobserved confounding variables, our ability to intervene on the system of interest in a modular way (i.e., without altering the rest of the causal system), and of course, the correct specification of the statistical model itself (Pearl, 2009; Robins, 2003; Eichler & Didelez, 2010 VanderWeele, 2015). As such we can understand the use of path-tracing and centrality measures as a first approximation to a possible underlying causal effect, an approximation which is seemingly valid under highly idealized conditions.

However, a well-known critique of DT-VAR models casts doubt on the veracity of such an interpretation even in such ideal conditions. This critique focuses on the property that lagged regression parameters exhibit *time-interval dependency*, hereby referred to as the *time-interval problem* (Gollob & Reichardt, 1987; Oud & Jansen, 2000; Reichardt, 2011; Voelkle et al., 2012; Deboeck & Preacher, 2016; Kuiper & Ryan, 2018).^2^ Gollob and Reichardt (1987) offer a classic example of this problem regarding the effect of taking aspirin on headache levels. This effect is negligible 2 min after ingestion, moderate after 30 min, strong after two hours and zero 24 hour later.

The phenomenon of time-interval dependency is a straightforward implication of assuming an underlying DT-VAR model, as can be shown with a simple example. Take it that the parameters that are introduced in Fig. 1a represent the lagged relationships of the Stress-Discomfort system based on measurements taken at *one-hour intervals*; we denote these parameters as $\varvec{\Phi }(\Delta t = 1)$. In theory, we could also have observed all variables in the Stress-Discomfort system at twice that rate, that is, at *half-hour intervals*. The path model for this is depicted in the left panel of Fig. 1b, where the half-hour measurements that could have been observed are depicted as *latent* variables (i.e., ${\varvec{Y}}(t = 1\frac{1}{2})$ and ${\varvec{Y}}(t = 2\frac{1}{2})$). The effects matrix relating the half-hour realizations of the process is denoted $\varvec{\Phi }(\Delta t = \frac{1}{2})$. From the time-series literature, it is known that the parameters of these two models are related by the expression

2 $$\begin{aligned}{}[\varvec{\Phi }\left( {1/2}\right) ]^2 = \varvec{\Phi }(1) \end{aligned}$$

that is, by squaring the matrix of parameters at the shorter interval, we obtain the parameters at twice that interval (Hamilton, 1994).^3^ It is important to note here that squaring a matrix is not equivalent to squaring the parameters of that matrix: instead, any given parameter in $\varvec{\Phi }(1)$ is a function of multiple parameters in $\varvec{\Phi }(\frac{1}{2})$. For instance, the cross-lagged parameter which regresses $Y_{4, \tau + 1}$ on $Y_{1,\tau }$ can be re-written in terms of the shorter-interval parameters as $\phi _{42}(1) = \phi _{22}(1/2)\phi _{42}(1/2) + \phi _{42}(1/2)\phi _{44}(1/2) + \phi _{12}(1/2)\phi _{41}(1/2) + \phi _{32}(1/2)\phi _{43}(1/2)$.

When we compare the dynamical network based on the one-hour and half-hour parameters (i.e., Fig. 1a, b, respectively), three consequences of the time-interval problem for network researchers become apparent. A first consequence is that networks based on different time-intervals can lead to seemingly contradictory conclusions regarding the sign, size and relative ordering of effects. For example, in the one-hour network, Stress and Anxiety both have positive lagged effects on Physical Discomfort, with the effect of Anxiety being slightly larger; yet, in the half-hour network, the corresponding lagged relations are both strongly negative, with the effect of Stress being larger (cf. Kuiper & Ryan, 2018). Since centrality measures are mere summaries of lagged parameters, this implies that different time-intervals between the observations are likely to lead to different centrality measures and, as a result, to different suggestions for intervention targets.

A second consequence of the time-interval problem is that, if data were obtained with unequal intervals and this is not accounted for, then the estimated parameters are a blend of the lagged relationships at different intervals present in the data. Although inserting missing observations can somewhat correct for unequal intervals (such as implemented in the DSEM module in Mplus; Asparouhov et al., 2018), the results of these techniques can at best only approximate the lagged parameters for a single target time-interval (De Haan-Rietdijk et al., 2017).

A third consequence of the time-interval problem is that the interpretation of any lagged parameter as a *direct* effect becomes questionable. Specifically, based on the relationship in Eq. (2), the lagged parameters of the one-hour network $\varvec{\Phi }(1)$ should be interpreted as *total* rather than *direct effects* (Deboeck & Preacher, 2016; Aalen et al., 2016).^4^ Take for example in the one-hour path model the cross-lagged relation from current Anxiety ($Y_{2,\tau }$) to Physical Discomfort an hour later ($Y_{4,\tau +1}$), controlling for current values of all other variables. This parameter ($\phi _{42}(1) = 0.077$) has a seemingly intuitive interpretation as a direct effect when we consider only observed values of the Stress-Discomfort system. However, when we examine how these variables are related to one another at half-hour intervals, we see that this relationship is in fact made up of a number of different pathways through latent values of our processes in between measurement occasions. These include direct paths ($Y_{2,\tau } \rightarrow Y_2(1\frac{1}{2}) \rightarrow Y_{4,\tau + 1}$ and $Y_{2,\tau } \rightarrow Y_4(1\frac{1}{2}) \rightarrow Y_{4,\tau + 1}$) as well as indirect paths through latent values of Stress ($Y_{2,\tau } \rightarrow Y_1(1\frac{1}{2}) \rightarrow Y_{4,\tau + 1}$) and Self-Consciousness ($Y_{2,\tau } \rightarrow Y_3(1\frac{1}{2}) \rightarrow Y_{4,\tau + 1}$).

Taken together, this shows current practice in dynamical network analysis—using summaries of DT-VAR parameters to find intervention targets—is flawed due to the time-interval problem. However, our presentation here also highlighted one potential solution to the time-interval problem: decomposing lagged relationships between observations into truly direct and indirect effects operating over a shorter time-interval. This decomposition opens up a new perspective on how lagged relationships should be interpreted, a perspective which we can use to explore time-interval dependency, and avoid coming to misleading or contradictory choices regarding intervention targets.

## A Continuous-Time Approach to Dynamical Network Analysis

In this section, we will present a Continuous-Time (CT) approach to dynamical network analysis, and discuss how it helps to overcome the time-interval problem and its consequences identified in the previous section. We will begin by introducing the basic notion behind CT models in terms of *stochastic differential equations*, and discuss how the parameters of that model can be interpreted as encoding moment-to-moment direct effects. Second, we introduce a new type of network representation to the psychological literature, encoding the sign and strength of these moment-to-moment relations, known as a weighted local dependence graph. Third, we describe how this CT model can equivalently be expressed as the CT-VAR model, which establishes the link between the DT-VAR model parameters and an underlying CT process. Finally, we describe the novel insights that are gained by using the CT network approach, and reflect on the implications of this approach for current practice in dynamical network analysis.

### Continuous-Time Processes and Differential Equations

In the previous section, we have shown how a single latent measurement wave between consecutive observations changes the way we should interpret DT-VAR parameters. Taking this approach one step further, it can be argued that for many psychological processes there can be *infinitely many* latent waves in-between two measurement occasions, and that such processes should be characterized as evolving *continuously* over time rather than in discrete “jumps” (cf. Boker, 2002; Coleman, 1968; Deboeck & Preacher, 2016; Driver et al., 2017; van Montfort et al., 2018; Ou et al., 2019; Oud & Jansen, 2000; Oravecz et al., 2011; Ryan et al., 2018; Voelkle et al., 2012). For example, it is reasonable to think that processes like stress and anxiety continue to vary in-between measurement occasions, and that, if those processes influence one another, they also do so in a continuous manner over time (for an extended discussion see Boker, 2002). Popular methods like experience sampling, which are based on measuring individuals at random points in time, seem to adhere to this notion that we are dealing with CT processes (at least while the participant is awake). Hence, it seems reasonable to suggest that many of the target processes being studied by dynamical network researchers in psychology, can be conceptualized as CT processes (e.g., Bringmann et al., 2013; Groen et al., 2020; Pe et al., 2015; Fisher & Boswell, 2016; Rubel et al., 2018; Bak et al., 2016).

**Fig. 2:**
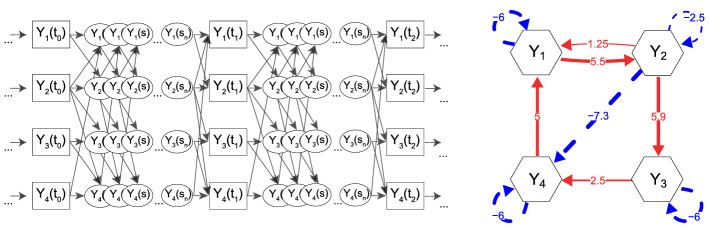
CT-VAR path model (left-hand side) and CT network in the form of a weighted local independence graph representing ${\varvec{A}}$ (right-hand side). In the path model, the latent variables and ellipses represent an infinite number of latent unobserved process values in-between measurement occasions, spaced an infinitesimally small time-interval apart; the presence of an arrow linking two variables in the path model denotes some nonzero dependency between them, conditional on all variables at the previous “wave,” that is, a local dependency. For the networks, the arrows represent auto- and cross-effect parameters of the drift matrix in a CT-VAR model. Solid red arrows denote positive parameters and dashed blue arrows represent negative parameters (Color figure online).

In SEM terms, we can represent a CT process as a path model in which there are infinitely many latent variable values in-between any two measurement occasions, spaced an infinitesimally small time-interval apart, as depicted in the left-hand panel of Fig. 2 (see also Singer, 2012; Deboeck & Preacher, 2016). Modeling CT processes is based on breaking down the relations between observed measurement waves into their fundamental building blocks, to obtain the truly direct lagged relationships operating over an infinitesimally small time-interval, which we will refer to as *moment-to-moment* effects. These continuous moment-to-moment dynamics are captured by *differential equation* models.

In the current paper, we will limit ourselves to considering a very simple type of differential equation model, known as a first-order stochastic differential equation, which can be thought of as the CT counterpart of a DT-VAR model which exhibits positive auto-regression.^5^ It can be written as

3 $$\begin{aligned} \frac{\mathrm{d}{\varvec{Y}}(t)}{\mathrm{\mathrm{d}}t} = {\varvec{A}}{\varvec{Y}}(t) + {\varvec{W}}(t) \end{aligned}$$

where $\frac{\mathrm{d}{\varvec{Y}}(t)}{\mathrm{d}t}$ on the left is the first derivative or the *rate of change* of the variables ${\varvec{Y}}$ at time *t* (denoted ${\varvec{Y}}(t)$). We can think of this derivative as being equivalent to a (scaled) *change score* ${\varvec{Y}}(t+s) - {\varvec{Y}}(t)$ over the shortest possible time-interval ($\lim s \rightarrow 0$). This derivative is dependent on the current value of the variables ${\varvec{Y}}(t)$, and the $p \times p$ matrix of regression parameters which relates these two is called the *drift matrix*, denoted ${\varvec{A}}$. The ${\varvec{W}}(t)$ term represents a Wiener process, a special kind of mean-zero white noise residual term (described in greater detail by, among others, Oud & Jansen, 2000; Voelkle et al., 2012; Voelkle & Oud, 2013).

Like the DT-VAR model, the stochastic differential equation has an equilibrium value, here defined by its mean of zero. While the Wiener process pushes the system away from this equilibrium, the drift parameters ${\varvec{A}}$ determine how the variables react to these shocks, eventually returning the system back to equilibrium over time (Strogatz, 2015; Ryan et al., 2018). The key difference between the two models is in *how* this behavior is encoded. The DT-VAR describes this behavior in terms of discrete jumps, current process values determining future process values some fixed time-interval later. The stochastic differential equation describes the same behavior in terms of moment-to-moment changes, current process values determining the instantaneous rate of change of each process, over the smallest imaginable interval.

### CT Networks and Interpretation

As the critical effects matrix in the differential equation model, we can interpret the drift parameters as representing truly *direct* moment-to-moment dependencies between our processes of interest. This interpretation of the drift matrix parameters makes them a natural choice to use as edges in a network-representation of the CT system. This type of network is known as a *local dependence graph* (Schweder, 1970; Aalen et al., 1980; Didelez, 2008) with *local* here referring to the idea that these relationships are locally spaced in time. As such, if there is a direct moment-to-moment relationship from $Y_i$ to $\mathrm{d}Y_j/\mathrm{d}t$ ($a_{ji} \ne 0$) we say that $Y_j$ is *locally dependent* on $Y_i$ and draw an arrow $Y_i \rightarrow Y_j$; if there is no such relationship ($a_{ji} = 0$) we say that $Y_j$ is locally independent of $Y_i$ and omit that arrow. By assigning a weight to these local dependencies based on the value of the drift parameters, we create a *weighted local dependence graph*, hereafter referred to as a CT network. The drift matrix parameters of the Stress-Discomfort system are plotted as a CT network on the right-hand side of Fig. 2, using hexagonal nodes to distinguish these from DT-VAR networks.

The interpretation of individual drift matrix parameters is similar to that of a change-score model from the time-series literature. For researchers familiar with DT-VAR models, however, some care should be taken when interpreting the diagonal parameters $a_{ii}$, known as *auto-effects*, which encode the relationship a variable has with its own rate of change. These auto-effects are typically negative, which ensures the system tends to move back toward its equilibrium as positive deviations are followed by negative changes, and negative deviations are followed by positive changes. Moreover, the auto-effects are not bounded, and can thus run from $-\infty$ (i.e., auto-regression close to 0), to 0 (i.e., auto-regression close to 1). In our example, we see a larger negative auto-effect for Stress ($a_{11} = -6$) than for Anxiety ($a_{22} = -2.5$), meaning Anxiety moves back toward equilibrium less quickly than Stress after a perturbation (for more details, see for example Oravecz et al., 2011; Ryan et al., 2018).

From a network perspective, what is likely to be of primary interest for applied researchers is the interpretation of the off-diagonal elements of the drift matrix ($a_{ji}, j \ne i$). These are the parameters we think of as direct moment-to-moment relationships between different processes. These off-diagonal parameters are also referred to as *cross-effects*, and have a very similar interpretation to cross-lagged parameters from a DT-VAR model: the negative cross-effect of Anxiety on the rate of change of Physical Discomfort ($a_{42} = -7.3$) means that an increase in the value of Anxiety will produce a decrease in the rate of change, and thus, the value of Physical Discomfort a moment later. The equivalence of these two statements can be seen by re-arranging the differential equation as an auto-regressive model over an infinitely small time-interval, as shown in Appendix B. As is the case for cross-lagged parameters, the higher the absolute value of the parameter, the greater the magnitude of the effect (for more details on the interpretation of these parameters, see also Oravecz et al., 2011; Ryan et al., 2018; Voelkle et al., 2012). When comparing this CT network to the DT networks presented in Fig. 1, we can see that there are many fewer direct dependencies. For instance, Stress has no direct moment-to-moment effect on Physical Discomfort ($a_{41} = 0$). Furthermore, the nonzero connections are all positive, apart from the strong negative effect of Anxiety on Physical Discomfort ($a_{42} = -7.3$). Hence, this raises the question where such differences between the two networks stem from.

### The Integral form: CT-VAR

The key to establishing a link between the CT and DT approaches is that the first-order stochastic differential equation defined above can also be expressed in its *integral form*. The latter is known as the CT-VAR or Ornstein–Uhlenbeck process (Oud & Jansen, 2000; Oravecz et al., 2009; Voelkle et al., 2012), and can be written as

4 $$\begin{aligned} {\varvec{Y}}(t_\tau )={\varvec{e}}^{{\varvec{A}} \Delta t_\tau } {\varvec{Y}}(t_{\tau -1}) + \varvec{\epsilon }(\Delta t_\tau ) \end{aligned}$$

where variables at the current measurement occasion ${\varvec{Y}}(t_\tau )$ are regressed on variables at the previous measurement occasion ${\varvec{Y}}(t_{\tau -1})$. Note that $\tau$ refers to the measurement occasion, whereas *t* refers to the actual time when this measurement took place. Hence, $\Delta t_\tau = t_{\tau } - t_{\tau -1}$ indicates the time-interval between two consecutive measurement occasions, which may differ across pairs of observations.

The above expression of the CT-VAR model is very similar to the DT-VAR model that was presented in Eq. (1). The variables are centered, and so an intercept term is again omitted, while the vector $\varvec{\epsilon }(\Delta t_\tau )$ contains the residuals, which are normally distributed with mean zero and variance-covariance matrix that is also a function of the time-interval (for more details, see Oud & Jansen, 2000; Voelkle et al., 2012; Voelkle & Oud, 2013). In place of the $\varvec{\Phi }$ matrix in Eq. (1), these lagged variables are related by the *matrix exponential* of the drift matrix multiplied by the time-interval ${\varvec{e}}^{{\varvec{A}} \Delta t_\tau }$. It follows from this that the key effects matrices from the CT- and DT-VAR model are related to each other by the expression

5 $$\begin{aligned} {\varvec{e}}^{{\varvec{A}} \Delta t} = ({\varvec{e}}^{{\varvec{A}}})^{\Delta t} = \varvec{\Phi }(\Delta t) \end{aligned}$$

which shows that the lagged parameters for any particular time-interval $\varvec{\Phi }(\Delta t)$ can be found by taking the matrix exponential of the moment-to-moment drift matrix ${\varvec{A}}$ to the power of the length of that time-interval $\Delta t$ (cf. Oud & Jansen, 2000; Voelkle et al., 2012). Recall that to relate the half-hour and one-hour parameter matrices in Eq. (2), we also took the matrix associated with the half-hour interval and raised it to the power two to get the matrix for a one-hour interval, a very similar operation to that defined here.

### Consequences of the CT-VAR for Dynamical Network Analysis

**Fig. 3:**
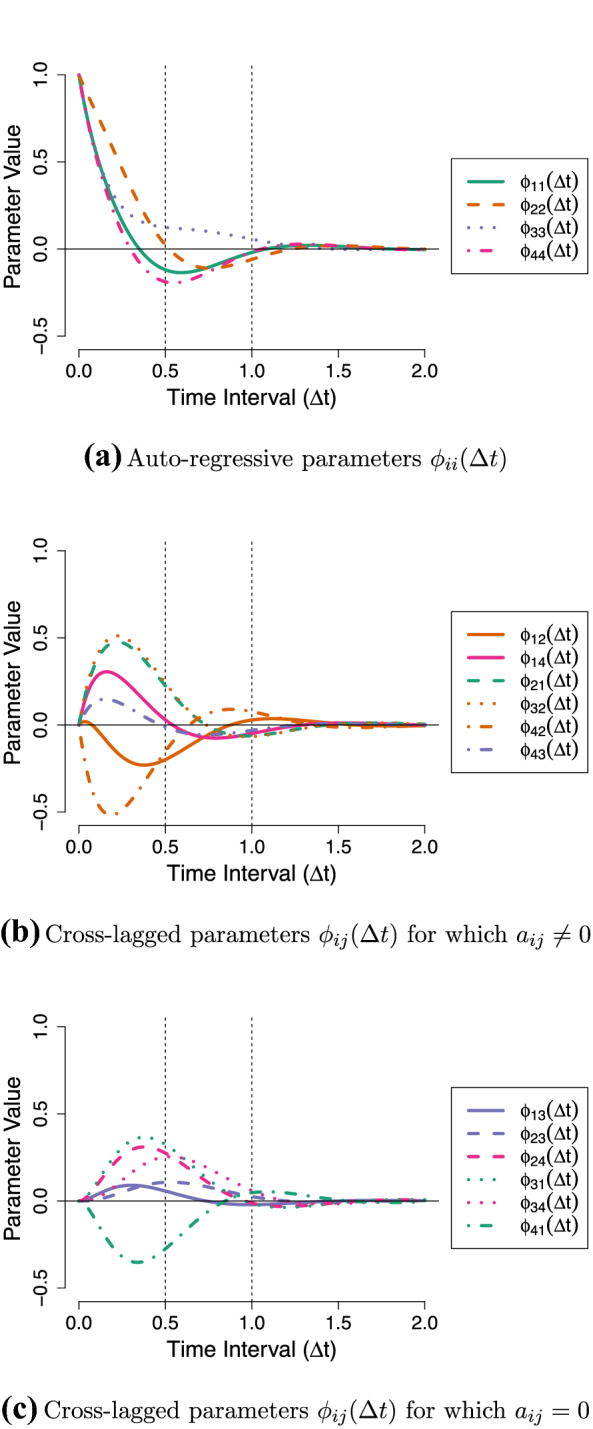
Lagged regression parameters as a function of the time-interval for the Stress-Discomfort system. Black dotted lines indicate the parameter values of the half-hour and one-hour networks in Fig. 1a, b.

The connection that the CT-VAR model establishes between the DT-VAR and differential equation parameters has a number of important implications. First, if we know the drift matrix ${\varvec{A}}$ we can use the above relationship to derive how the DT lagged parameters are expected to evolve as a function of the time-interval. Figure 3 depicts the lagged relationships of the Stress-Discomfort system over a range of zero to two hours. It shows that the lagged parameters change continuously and smoothly. It also shows that the parameters reach their peak value at different time-intervals $\Delta t$, and that some parameters even change sign (positive/negative) over $\Delta t$. For instance, from Fig. 3b we can see that the effect of Anxiety on Physical Discomfort ($\phi _{42}(\Delta t)$) is strongly negative at very short intervals (around $\Delta t = 0.2$) before becoming weakly positive at longer intervals (from $\Delta t = 0.6$ to 1.2). In comparison, the peak effect of Anxiety on Stress ($\phi _{12}(\Delta t)$) occurs both at a longer time-interval and with a smaller magnitude. This information yields novel insights into the underlying dynamics which cannot be obtained by inspecting a single set of DT-VAR parameters, nor can it be easily read off from the local dependencies in the CT network.

**Fig. 4:**
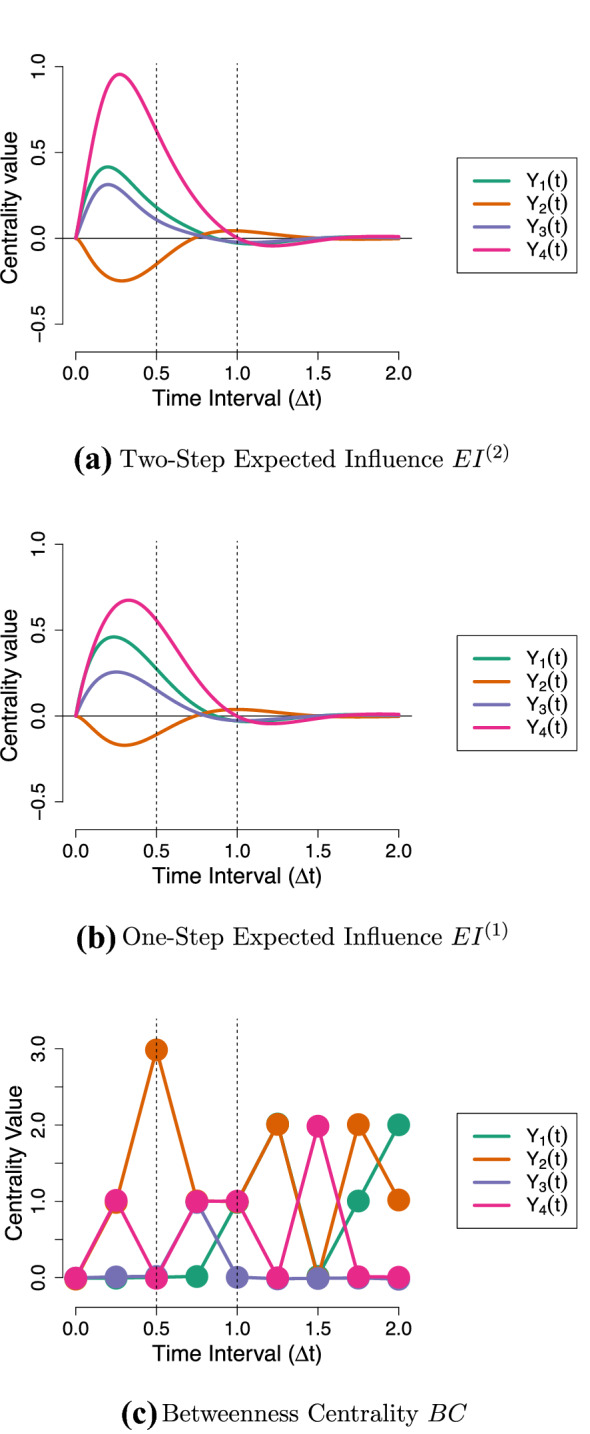
Centrality measures as a function of the time-interval for the Stress-Discomfort system. Black dotted lines indicate the centrality values of the half-hour and one-hour networks in Fig. 1a, b.

Second, the time-interval dependency of the lagged parameters shown above also implies that the network will change as we consider different time-intervals (see the Online Supplementary Materials 1 for an animation of this). As a result, the centrality measures—which are based on the network—also change as a function of the time-interval. This is shown in Fig. 4, where we see that rather different conclusions regarding intervention targets are liable to be made depending on the time-interval under consideration.

Third, it becomes clear that the lagged regression parameters at *any* time-interval $\Delta t$ should be interpreted as total rather than direct effects (Deboeck & Preacher, 2016). When the drift matrix contains the truly direct relationships, then the matrix exponent, as in both Eqs. (2) and (5), should be understood as a path-tracing operation (see Appendix B for an elaboration on how the matrix exponential relationship can be derived by re-arranging the first-order stochastic differential equation, and applying a path-tracing operation through $\lim n \rightarrow \infty$ latent measurement waves). This interpretation also explains the greater density of the DT-VAR networks in comparison to the CT networks, as the former is a sum of the direct and indirect effects defined by the latter over a particular time-interval. Yet, it is impossible to tell to what extent these are direct or indirect effects without knowledge or an estimate of the underlying drift matrix parameters.

### Conclusion

To summarize, the CT network approach outlined above has three major strengths, that is: (a) it allows for an elegant treatment of unequal time-intervals between observations in experience sampling data; (b) it introduces a new way to conceptualize a network of direct dynamic relationships between processes; and (c) it allows us to gain important new insights into the underlying process by exploring how lagged relationships are expected to vary and evolve as a function of the time-interval.

Deboeck and Preacher (2016) and Aalen et al. (2016) have already provided path-tracing rules for CT models with three variables and a lower-triangular drift matrix. We extend these rules to the general multivariate case without those constraints on the drift matrix in Appendix C, with accompanying *R* functions in Online Supplementary Materials 2 and in an *R* package *ctnet* available to download from the github page of the first author.^6^ This makes it possible for researchers to calculate any direct, indirect or total effect of interest from a CT model. However, as we have established, a core interest of dynamical network researchers is to use their estimated models to inform intervention targets. In the following section, we address some ways in which this could be done, primarily through the development of new centrality measures for CT networks.

## Interventions and Centrality for CT Networks

The use of generic centrality measures to identify intervention targets has been frequently critiqued in the network literature, most notably because the connection between centrality measures and interventions in any particular system is typically unclear (Borgatti, 2005; van Elteren & Quax, 2019; Dablander & Hinne, 2019). Clearly, to be able to choose an optimal intervention target we need to know what type of intervention we can apply and what type of effect we want to see in a particular type of system. For centrality measures to be useful for this purpose, they must be clearly defined in those terms.

In the following, we will take a first, highly simplified and idealized approach to the identification of intervention targets, assuming that our causal system is modular, that there are no unobserved confounding variables, and that a CT-VAR model forms an appropriate model of the system. Based on this, we will discuss two types of simple intervention that we could apply to a dynamic system, inspired by the concept of variable interventions from the causal inference literature (Pearl, 2009). Subsequently, we will show how CT path-specific effects can be conceptualized as describing the effects of these different types of interventions, and then use these to define new centrality measures that have a clear interpretation in terms of interventions and the change they produce. Finally, we will consider what other types of changes we might hope to bring about in a dynamic system.

### Pulse and Press Interventions

One of the fundamental conceptual building blocks of modern causal inference is the notion of an *intervention*. In the framework of Pearl (2009), an intervention is defined as an operation by which we manipulate the value of a *variable* in our causal system. This intervention is denoted using the *do*-operator, with $do(X = 1)$ denoting that we intervene to set the variable *X* to a constant value of one. In this paper, we will consider two different basic types of *do*-operation, reflecting two of the most basic types of intervention often discussed in relation to dynamical systems (Bender et al., 1984).

**Fig. 5:**
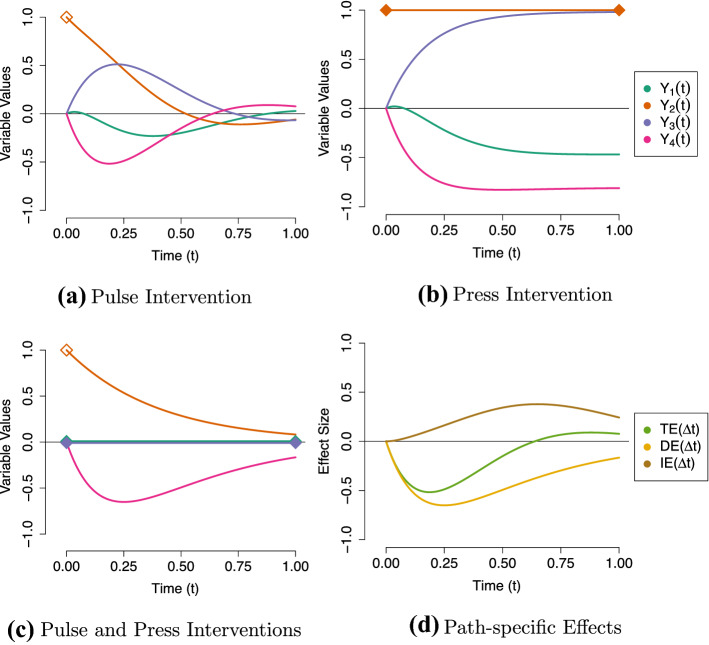
Illustration of pulse (denoted by an empty diamond) and press (denoted by a filled diamond) interventions and their relationship with total effects (TE), direct effects (DE) and indirect effects (IE). **a** Shows the effect of a pulse intervention on Anxiety ($Y_2(t)$) on the values of Stress ($Y_1(t)$), Self-Consciousness ($Y_3(t)$) and Physical Discomfort ($Y_4(t)$). **b** Shows a corresponding press intervention on Anxiety. **c** shows a combination of pulse intervention to Anxiety and press interventions to keep Stress and Self-Consciousness fixed at their equilibrium of zero. The consequence of this combination of interventions **d** shows the total, direct and indirect effect of Anxiety on Physical Discomfort. The total effect (${\textit{TE}}(\Delta t)$) is simply the trajectory of Physical Discomfort in (**a**). The direct effect (${\textit{DE}}(\Delta t)$) is the trajectory of Physical Discomfort in (**c**). The indirect effect (${\textit{IE}}(\Delta t)$) is the difference between those two trajectories (${\textit{TE}}(\Delta t) - {\textit{DE}}(\Delta t)$).

A *pulse* intervention is an operation by which we change the value of a variable at one particular point in time. Taking the Stress-Discomfort system as an example, we can imagine that it is possible to induce a momentarily high experience of Anxiety in our participant (for instance by making the participant view a unpleasant photograph, a manipulation which has been shown to increase state anxiety in laboratory studies; Richards & Whittaker, 1990; Richards et al., 1992). Using the *do* operator, we would denote such an intervention $do(Y_2(t) = 1)$ meaning we intervene to set Anxiety to a value of 1 at time *t*. The effect of this pulse intervention on the other processes in the system depends on the time since that impulse was applied; hence, we can visualize the effect of this intervention by plotting the expected trajectories of the different variables in our system.^7^ Figure 5a shows the effect that an initial intervention on Anxiety has on our Stress-Discomfort system: the other three variables leave their equilibrium, and eventually, the effect of the intervention fades and all variables return to their resting state.

A second type of intervention is the *press* intervention, which consists of changing the value a variable over an *interval* of time. For example, we may produce a longer-lasting state of high-anxiety by having participants prepare to give a public speech (e.g., Moscovitch et al., 2010; Azevedo et al., 2017), or induce longer-lasting changes in stress levels by prompting participants to engage in mindfulness meditation (e.g., Hoge et al., 2013). Using the *do*-operator, we denote such an intervention for the time-interval $\Delta t$ as $do(\overline{Y_2(t+\Delta t)}=1)$ (i.e., intervening to set Anxiety to the value 1 starting from time *t* and lasting $\Delta t$). The effect of this press intervention is shown in Fig. 5b: we see that the other three variables leave their equilibrium, and that they eventually settle to a new equilibrium. They will only return to their old equilibrium value if the intervention is removed. Of course, the effect of this press intervention depends on the value the manipulated variable is set to: if the intervened-on variable takes on its equilibrium value, no new equilibrium will appear.

### Path-Specific Effects and Interventions

We can show that CT path-specific effects, originally described by Deboeck and Preacher (2016) and Aalen et al. (2016) and generalized in Appendix C, describe the effect on a target variable of either a pulse intervention, or a combination of pulse and press interventions. The effects themselves are expressed as a *difference in expected value*, with this difference contrasting two sets of intervention values.^8^

First, a CT total effect simply describes the effect that applying a pulse intervention has on some other variable in the system. In Fig. 5a, we already saw the effect of setting Anxiety to a value of one, in that it pushed the other variables in the system away from their equilibrium. If we instead set Anxiety to its equilibrium value, $do(Y_2(t) = 0)$, none of the variables would move away from equilibrium. Let’s say we are primarily interested in the effect of pulses to Anxiety on the expected value of Physical Discomfort some time $\Delta t$ later. We define this as the total effect6To be able to actually compute the above expression, we need to plug in a model for the expected values. Here, we use the CT-VAR model, which results in

7 $$\begin{aligned} {\textit{TE}}_{24}(\Delta t) = {{\varvec{e}}^{{\varvec{A}} \Delta t}} _{\left[ 42\right] } \end{aligned}$$

where ${{\varvec{e}}^{{\varvec{A}} \Delta t}} _{[42]}$ denotes the parameter in the fourth row, second column of ${\varvec{e}}^{{\varvec{A}} \Delta t}$. This expression for the total effect is actually identical to the path-tracing definition of a total effect given by Deboeck and Preacher (2016) and Aalen et al. (2016) (for details, see Appendices C and D).

Second, CT direct effects simply described the consequence of a *pair* of interventions: a pulse intervention to *change* the cause variable, just as we did for the total effect, and another press intervention to *keep fixed* one or more mediating variables. Take for example the effect of applying a pulse to increase Anxiety, while simultaneously applying a press intervention to keep Stress and Self-Consciousness fixed at equilibrium. This is visualized by the trajectories in Fig. 5c. As before, Anxiety starts at a high level—as it was set to 1—and dissipates back to equilibrium. Because Stress and Self-Consciousness are kept fixed at all moments in time, Physical Discomfort is pushed even further from equilibrium than before; by press-intervening on the mediators, we no longer activate the compensating positive feedback loops that are present in the total effect (see also Fig. 2). We can express this direct effect as the difference between two conditional expectations8By plugging in the CT-VAR parameters for each conditional expectation, we can express the effect of this intervention as9where ${\varvec{A}}^{(D[-1,-3])}$ denotes the drift matrix in which the indirect pathway parameters that link Anxiety to Physical Discomfort are set to zero (that is, $a_{12}=a_{32}=a_{43}=0$). Hence, in this drift matrix only the *direct links* between Anxiety and Physical Discomfort are retained (see Appendix D for a proof). Again, we can see that this expression is exactly equivalent to the path-tracing definition of a direct effect described in Appendix C.

Finally, the indirect effect ${\textit{IE}}(\Delta t)$ describes how the effect of applying a pulse to $Y_i(t)$ *changes* when we also press-intervene to keep the mediator(s) $Y_k$ fixed. Suppose we are interested in the mediating roles that both Stress and Self-Consciousness play in the effect of Anxiety on Physical Discomfort. To quantify this, we would define the indirect effect of Anxiety on Physical Discomfort (relative to Stress and Self-Consciousness) as10The indirect effect is thus the difference in value of Physical Discomfort at a particular time-interval *t* in Fig. 5a (i.e., the total effect ${\textit{TE}}(\Delta t)$), and the value at the same time-interval in Fig. 5c (i.e., the direct effect ${\textit{DE}}(\Delta t)$). Each of the total, direct and indirect effects are shown together in Fig. 5d. If the indirect effect is *positive*, it means that ${\textit{TE}} > {\textit{DE}}$, that is, applying a press intervention on the mediators *decreases* the effect of the pulse intervention. If the indirect effect is *negative* (${\textit{TE}} < {\textit{DE}}$) the press intervention *increases* the effect of the pulse intervention. Of course, when giving any further substantive interpretation, careful attention should be paid to the signs of the component direct and total effects. In this case, keeping Stress and Self-Consciousness both fixed makes Physical Discomfort take on a stronger negative value at shorter intervals, and the indirect effect quantifies that difference. As such, this indirect effect describes the mediating role of the variables Stress and Self-Consciousness combined. We can express this indirect effect using the expressions we found before for the total and the direct effects in terms of the CT-VAR parameters. This gives us11It follows that the effect of this combination of interventions is equivalent to the path-tracing definition of the indirect effect (see Appendix C).

### Centrality Measures to Identify Intervention Targets

Having established the link between CT path-specific effects and highly idealized interventions in the dynamic system, we here propose two new centrality measures for CT networks. Each centrality measure is explicitly defined as a summary of a path-specific effect, and as such, as a kind of network-wide summary of the consequences of a particular intervention. This means that these centrality measures are functions of the time-interval and have a clear link to a particular type of variable intervention: the first and second measure can be used to identify the optimal target for a pulse and press, respectively.

#### CT Total Effect Centrality

We define our first new centrality measure as the *Total Effect Centrality* (${\textit{TEC}}$) of a variable, which can be calculated by summing the total effect of $Y_i(t)$ on all other variables, at a particular time-interval

12 $$\begin{aligned} {\textit{TEC}}_{i}(\Delta t) = \sum _{j\ne i}^{p} {\textit{TE}}_{ij}(\Delta t). \end{aligned}$$

Hence, we sum over all the total effects of $Y_i$ on other variables in the network (excluding $Y_i$ itself). The ${\textit{TEC}}$ thus summarizes the effect of an impulse intervention to change $Y_i(t)$, on the system as a whole, that is, the cumulative effect on the network, some time-interval $\Delta t$ later. Since we explicitly make this centrality measure a function of the time-interval, we can examine how the cumulative effect of this intervention evolves following the pulse.

**Fig. 6:**
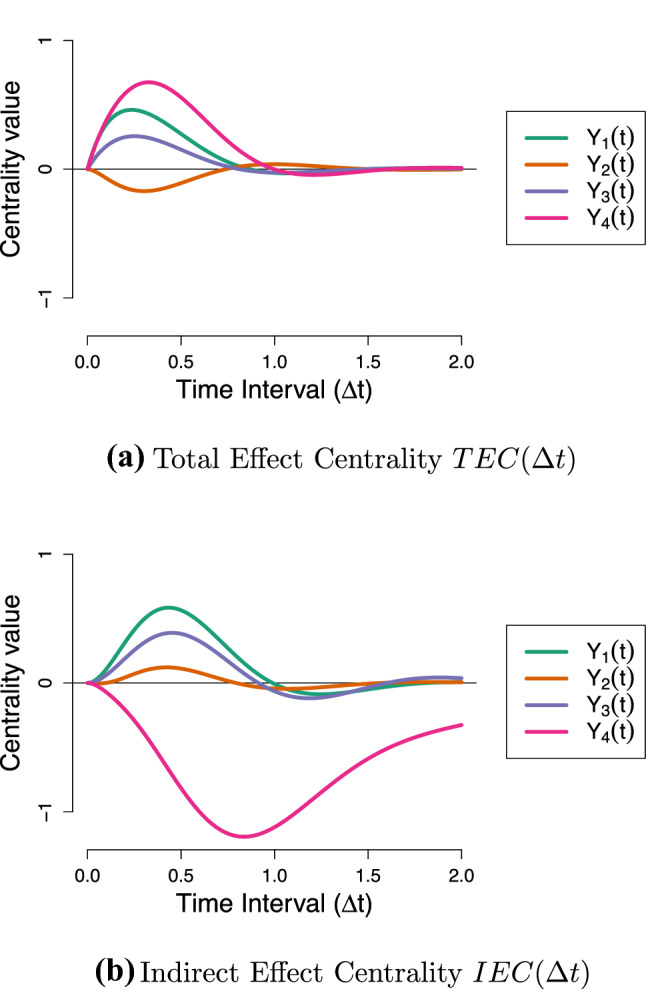
Illustration of the new total and indirect centrality measures for CT networks, applied to the Stress-Discomfort system.

Figure 6a shows the ${\textit{TEC}}$ of each variable in the Stress-Discomfort system over a range of intervals, from $\Delta t = 0$ to $\Delta t = 1.5$. From this, we can see that at short intervals, pulse intervention to increase Physical Discomfort has the biggest cumulative effect on the network: overall, this intervention on Physical Discomfort results in the other variables increasing in value over the next half an hour or so, before eventually the effect of this intervention fades away. Notably, an intervention to increase Anxiety has a weak net negative effect on the system at shorter intervals, and a weak net positive effect at longer intervals: we would expect this based on our visualization of that intervention in Fig. 5a, where a pulse to Anxiety resulted in Stress and Physical Discomfort taking on negative values at short intervals.

The ${\textit{TEC}}$ measure allows us to see that, for this system, Physical Discomfort is the optimal target for a pulse intervention, assuming that we can set Physical Discomfort to a low or negative value (e.g., $do(Y_4(t)) = -1$). Such an intervention would be expected to result in a short-lived decrease in value of the other processes in the model. Note that, as a consequence of DT-VAR parameters reflecting total effects from a CT perspective the ${\textit{TEC}}$ measure is actually equivalent to calculating the first-order expected influence measure using $\varvec{\Phi }(\Delta t)$ over a range of values for $\Delta t$ (as depicted in Fig. 6b). Recall however that the latter is erroneously interpreted as a measure of direct rather than total influence.

#### CT Indirect Effect Centrality

The second centrality measure we propose quantifies the role a particular variable plays *as a mediator* of other relationships between variables in the network. To define this measure, we use the indirect effect measure described in Eq. (10). Recall that the CT indirect effect captures the *change* in the effect of pulses to $Y_j(t)$ on the value of $Y_k(t+\Delta t)$, if we press-intervene to keep the mediator $Y_i$ fixed at every moment in time (). Hence, we define the *Indirect Effect Centrality* (${\textit{IEC}}$) of a *mediator variable* $Y_i$ as
13that is, it represents the sum of all possible indirect effects between different pairs of variables $Y_j(t)$ and $Y_k(t+\Delta t)$, in which $Y_i$ serves as the only mediator. Note here that the notation is chosen to reflect that the ${\textit{IEC}}$ is a property of a mediator, instead of a property of one particular cause-effect relationship. The summation denotes that we omit auto-regressive relationships ($j \ne k$) and pairs of variables where the mediator is either the cause or effect variable ($j \ne i$ and $k \ne i$).

The ${\textit{IEC}}$ quantifies how a press intervention on $Y_i$ changes the effects that other variables have on each other. This may be a very useful concept in clinical practice. For instance, suppose that in our Stress-Discomfort system we want to avoid a high value on all four variables as much as possible. The current measure can be used to determine which of these variables is most important in terms of mediating the effects of one variable on another in the system, such that by intervening on this variable, these indirect paths become blocked and the flow of activation from one variable to another is (partly) interrupted.

Figure 6c shows the ${\textit{IEC}}$ of each variable over a range of intervals. It shows that Physical Discomfort has the strongest indirect effect centrality in absolute terms. A strong negative value of ${\textit{IEC}}$ means that keeping Physical Discomfort fixed at an equilibrium value actually *increases* the effects of pulses to other variables on each other, since the component direct effects are greater than the corresponding total effects. This happens because Physical Discomfort plays a key role in the only *negative* feedback loop in the network: since an increase in Anxiety actually *decreases* Physical Discomfort ($a_{42} = - 7.3$), the total effect of Anxiety on Stress is less strong than its direct effect. If, however, we intervene to keep Physical Discomfort fixed, then this negative compensating effect is not activated, meaning an increase to Anxiety in fact has a *greater* effect on the network as a whole. Stress has the largest positive ${\textit{IEC}}$, meaning that keeping Stress fixed *decreases* the effects of other variables on one another.

From this, we would conclude that we should choose *Stress* as a target for a press intervention, as it decreases the short-term impact of other variables in the network on each other. Moreover, we should avoid applying a press intervention on Physical Discomfort: such an intervention would in fact increase the strength of positive relationships between the other variables.

### Other Ways of Identifying Intervention Targets

We have presented a selection of measures for CT models that have a clear conceptual link with path-tracing and centrality measures as well as hypothetical variable interventions. The centrality measures we introduce summarize both total and indirect effects in a CT network, but we did not develop a centrality metric based on the direct effects: such a measure would require us to add different direct effects such that each requires a different set of variables to be held constant, and so, are not directly informative about any particular variable intervention.^9^

The total and indirect measures introduced here allow us, respectively, to choose a target for a pulse intervention which will result in the largest “shock” to the system, and choose a target for a press intervention which will result in the largest change in how other variables activate one another. As is typical for psychological network analyses, we have chosen variables such that positive values have a negative connotation: we want to avoid high values of Stress, Anxiety, Self-Consciousness and Physical Discomfort, so we want to apply negative-valued pulses, and in general, press interventions that lower the degree to which variables activate one another. We believe that our approach of visualizing the effects of these interventions, and how they change depending on the time-interval, is the most informative approach for researchers, but these measures can also be summarized (for example, by averaging or taking the “area under the curve” over a particular time-interval).

Of course, in addition to the centrality measures described above, researchers may wish to apply interventions in order to affect different types of change in the system. For example, we may wish to find a press intervention such that the equilibrium values of the other variables in the system change in a particular way, as we saw in Fig. 5b. We provide *R* code in Online Supplementary Materials 2 (and accompanying *R* package *ctnet*) which can be used to simulate the effect of different press interventions on the equilibrium positions and the stability of a system (see also Appendix D.3). Of course, many different types of interventions in a system are also possible: Driver and Voelkle (2018b) focus on different interventions than the ones considered here, and show how these can be simulated from CT models. However, from a causal modeling perspective, we believe that, while any operation on a model’s parameters can be defined in principle, typically if we want to learn about the effects of those interventions, or understand how we might bring about those changes in the system, we will likely need to define those manipulations in terms of interventions on variables in our causal model, as we have done in the current paper.

## Empirical Example

In this section, we illustrate the application of CT network analysis, as developed in the previous sections, to an empirical ESM dataset. Throughout, we compare the estimated CT network structure and centrality measures to their commonly used DT equivalents, based on estimating a DT-VAR model. The latter ignores the unequal time-intervals between observations, and results in total rather than direct effects; it is therefore expected to lead to different conclusions than the CT-VAR model. All models here were estimated using a maximum-likelihood approach based on *stan* (Gelman et al., 2015) functionality in the *ctsem* package (Driver et al., 2017). The *R* code to reproduce all analyses shown here is provided in Online Supplementary Materials 2.

### Data

**Fig. 7:**
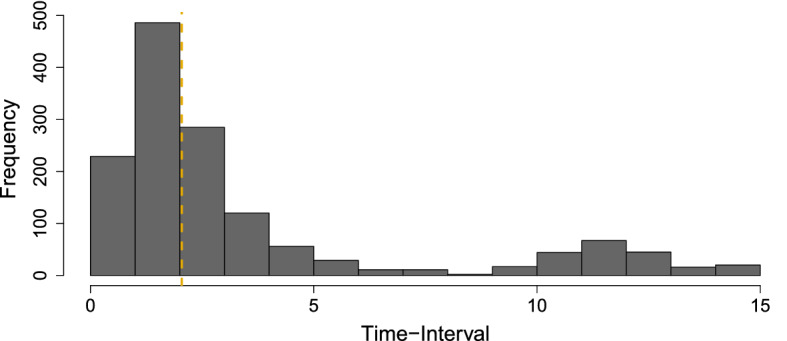
Histogram showing the distribution of time-intervals between subsequent measurement occasions in the empirical dataset.

To illustrate the CT network approach, we use a single-subject open-source ESM dataset.^10^ A subset of this dataset was originally the subject of a DT-VAR network analysis by Wichers et al. (2016) and is described in full in Kossakowski et al. (2017). For illustrative purposes, we chose to fit a CT-VAR model using four ESM items measured on a 7-point Likert scale from low to high agreement: Self-Doubt (S; “I doubt myself”), Fatigue (F; “I am tired”), Irritated (I; “I feel irritated”) and Restless (R; “I feel restless”). Prior to analysis the variables were standardized. A subset of the full time series is used, consisting of 1476 measurements taken over 239 consecutive days (reflecting a period preceding a blinded medication reduction). The randomized sampling scheme results in time-intervals between consecutive measurements ranging from 13.5 min to 42.1 h with a median of 2.04 h. Figure 7 displays the distribution of time-intervals up to the 97.5 percentile. Inferences made from the CT model beyond the time-intervals used in data collection represent a form of model extrapolation and should be approached with caution, so we will focus our analysis in the following on the observed time-interval range ($\Delta t = 0$ to $\Delta t = 5$).

### Estimated Networks

**Fig. 8:**
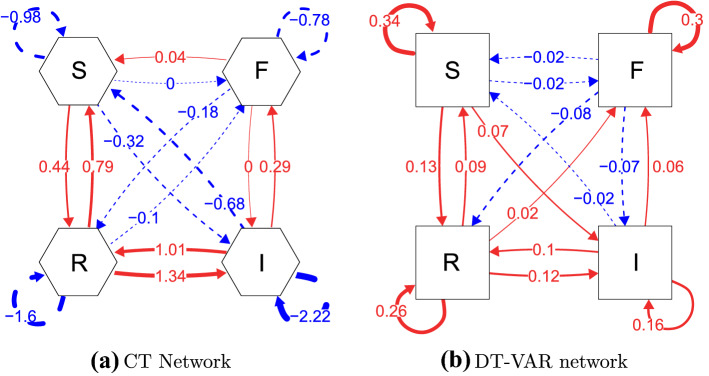
Estimated networks based on the CT-VAR and DT-VAR analysis of empirical data, respectively. S (Self-Doubt); F (Fatigue); I (Irritated) and R (Restless). **a** Shows a weighted local dependence graph based on $\varvec{\hat{A}}$ while **b** shows a DT-VAR network based on $\varvec{\hat{\Phi }}$. In the latter case, time-interval information was ignored in estimation.

As a first step, we can inspect and interpret the estimated drift matrix parameters $\varvec{\hat{A}}$. These are displayed as a CT network in Fig. 8a, with accompanying confidence intervals for these parameters given in Appendix E. From this, we can see that, for instance, Irritated and Restless have the highest auto-effects, implying that, given a shock to the system, these processes would be expected to return to baseline quicker than feelings of Self-Doubt or Fatigue. Furthermore, the strongest cross-effects in the network are the positive reinforcing relationships between Irritated and Restless: Feeling Irritated is likely to increase your feelings of Restlessness a moment later, and vice versa.

The estimated DT-VAR model displayed in Fig. 8b shows a somewhat similar pattern of relationships between processes, but there are some key differences. Most notably, the signs of several cross-lagged relationships are different from the corresponding drift matrix estimates: there are positive cross-lagged effects from Self-Doubt to Irritated, from Restless to Fatigue and from Fatigue to Self-Doubt. Furthermore, the relative ordering of cross-lagged parameters is different than that of the drift matrix parameters.

### Exploring Time-Interval Dependency

**Fig. 9:**
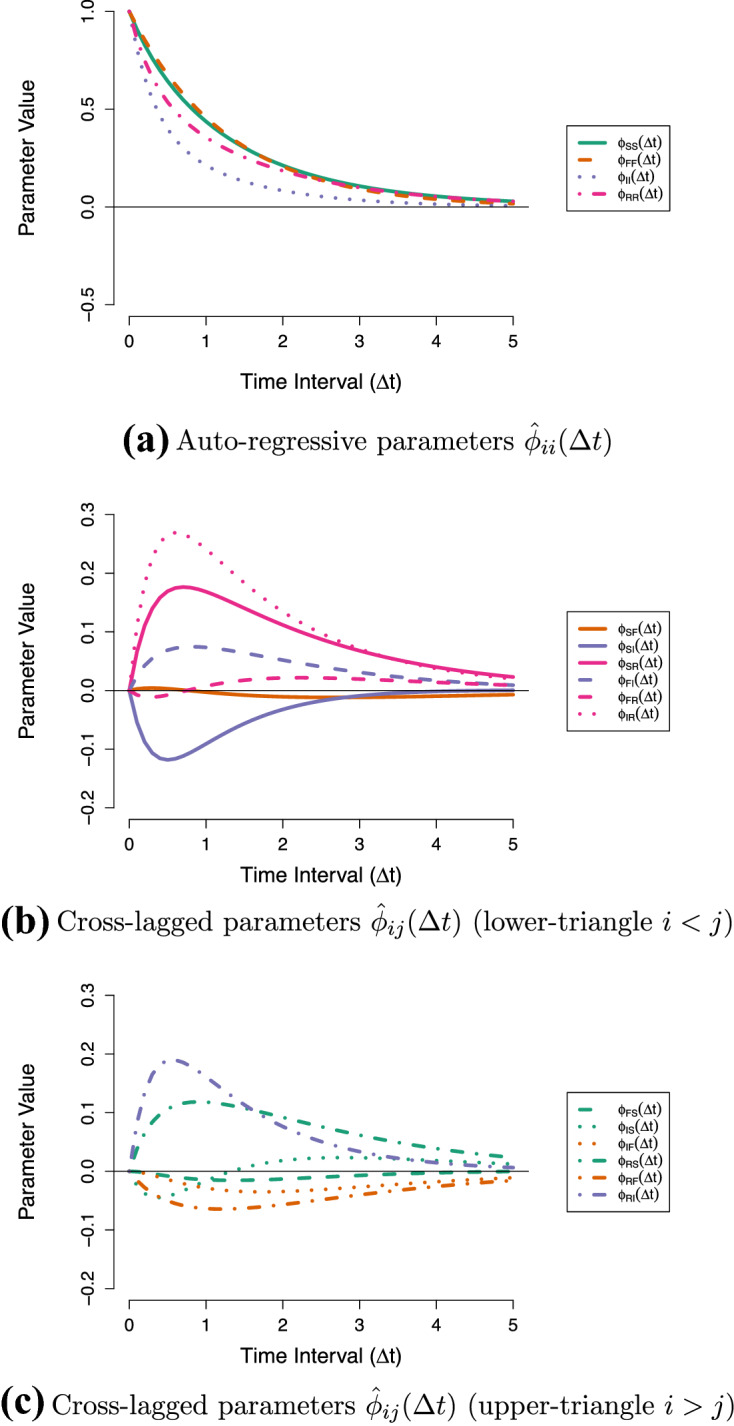
Expected lagged regression parameter values as a function of the time-interval, derived from drift matrix estimated from empirical data. Here, the subscripts S (Self-Doubt), F (Fatigue), I (Irritated) and R (Restless) refer to the first, second, third and fourth dimension of the drift matrix, respectively.

To see how the system evolves over time, we can use the drift matrix to derive how we would expect the lagged regression parameters to change as a function of the time-interval (that is, we use $\varvec{\hat{A}}$ to derive $\varvec{\hat{\Phi }}(\Delta t)$). This is shown in Fig. 9.

First, we can see that the auto-regressive relationships in Fig. 9a are quite similar over a range of intervals. Second, from Fig. 9b, c we can see that most cross-lagged relationships are expected to reach their peak between a half hour ($\Delta t =0.5$) and one and a half hours ($\Delta t = 1.5$). Third, we can see that the ordering of some effects changes depending on the interval: for instance, panel (c) shows that at short intervals the effect of Irritated on Restless ($\phi _{RI}(\Delta t)$) is larger than that of Self-Doubt on Restless ($\phi _{RS}(\Delta t)$), but at longer intervals ($\Delta t > 1.5$) this is reversed. Fourth, we can see from Fig. 9c that the cross-lagged effect from Self-Doubt to Irritated ($\hat{\phi }_{IS}(\Delta t)$) changes sign over $\Delta t$: it is negative at very short time-intervals, and becomes positive at longer intervals (around $\Delta t = 1$). This pattern results from the direct negative moment-to-moment dependency ($\hat{a}_{IS} = -0.47$) which is dominant at shorter intervals, while the effect at longer intervals is mostly driven by positive *indirect* relations through Restless.

### Centrality Analysis

**Fig. 10:**
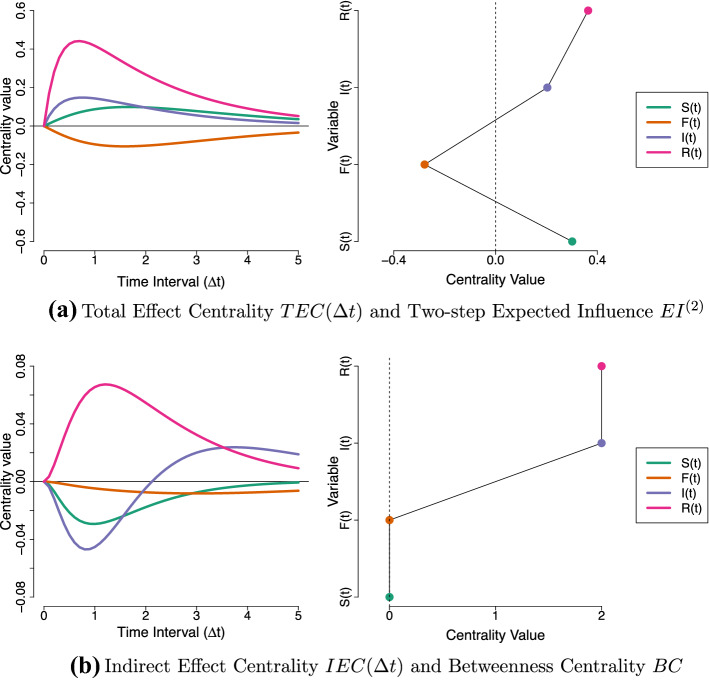
Total, Direct and Indirect centrality metrics for S(*t*) (Self-Doubt), F(*t*) (Fatigue), I(*t*) (Irritated) and R(*t*) (Restless) based on the CT Network (left-hand side) and DT-VAR Network (right-hand side), respectively.

The values of the estimated CT centrality metrics are shown in Fig. 10. For comparison, we also include the corresponding centrality measures one would compute based on the DT-VAR results. Figure 10a shows that Restless has the highest total effect centrality value over the entire range of intervals from zero to five hours. This implies that (making the same idealizing assumptions outlined in previous sections) it is the optimal target for a pulse intervention: we would expect such an intervention to have a large impact on the network as a whole, peaking around $\Delta t = 0.75$ after the impulse. Since this centrality measure is positive in this interval range, we would recommend applying a negative impulse to Restless, which will lead to a decrease (rather than increase) of the other variables in the network.

From Fig. 10b, we see that Restless alone has a high positive indirect effect centrality value, indicating that applying a press intervention to Restless is expected to *decrease* the degree to which positive impulses in one part of the network results in positive activation in other parts of the network. Furthermore, we can see that a press intervention on Irritation is expected to actually increase activation levels at short intervals (i.e., make the other variables more-connected), and decrease them at longer intervals (i.e., making them less-connected). From this, we would conclude that Restless appears to be the most attractive target for a press intervention, and that press interventions on Tired and Self-Doubt should be avoided.

In the right-hand side of Fig. 10a, b, we show the related DT centrality measures based on the estimated DT-VAR parameters. In Fig. 10a, we see that Restless has the highest two-step expected influence scores, closely followed by Irritated and Self-Doubt. In Fig. 10b, we see that Restless and Irritated have the joint highest Betweenness scores. In this case, applying current practice, we may very well have concluded that Restless and Irritated are candidate intervention targets. However, only with the CT approach developed here could we make specific recommendations regarding the type of intervention to apply, understand the effect such an intervention is expected to have and describe the subtle differences in intervention effects across short and longer time-intervals we would expect to see.

## Discussion

In this paper, we have introduced a new method for dynamical network analysis based on the use of CT models. We have shown that, from a CT perspective, the use of DT-VAR models for network analysis is potentially highly problematic: DT-VAR parameters are time-interval dependent, should not be interpreted as direct effects, and yield centrality measures which may lead to the selection of sub-optimal intervention targets. In contrast, the CT approach has several advantages: CT-VAR models aim to estimate truly direct moment-to-moment dependencies, and adequately handle unequal time-intervals between observations. The developments in this paper allow researchers to utilize CT models in a network setting: we have showed how to represent CT parameters as a dynamical network, explore time-interval dependency, and use the newly developed CT centrality measures to choose where to apply a pulse or press intervention under highly idealized conditions.

### Limitations

While we have tried to build a case for CT modeling, of course it should not be considered a panacea. CT models do not solve the problem of unobserved confounding, a substantial threat to the causal interpretation of models and identification of intervention effects from observational data in all psychological settings. In particular, we suspect that the effects of press interventions may prove difficult to identify from observational data, since the calculation of their effect relies heavily on correct specification of the CT model. However, by defining our targets of inference in terms of variable interventions, we can in principle connect these measures with the modern causal inference literature, much of which is concerned with deriving the conditions under which such interventions are identifiable (Pearl, 2009). Of course, this task is far from trivial, and much more research is needed to derive these conditions (for examples of work in this direction, see Eichler & Didelez, 2010; Didelez, 2015, 2019; Sokol, 2013; Gische et al., 2020). Drawing causal conclusions from statistical models should always be approached with great caution. We consider the developments presented in the current paper only as a first step in the right direction, providing clarity about what (CT-)VAR models can tell us about variable interventions in a best-case scenario. Ultimately, any approach to deriving intervention targets from statistical models must be evaluated in terms of its success in predicting the effect of actual interventions, necessitating the collection of the relevant experimental data.

From a theoretical standpoint, one may question the validity of treating psychological processes as evolving continuously over time. For instance, while we can measure variables like stress throughout waking life, this leaves open the question of how to treat periods of sleep: does the process continue to change continuously during these episodes, do the variables stay fixed, do they temporarily cease to exist (representing a discontinuity) and/or do they simply “reset” after sleep? While this is a valid concern, in the current case we believe that the practical implications of periods of sleep, at least, are not severe. Since the CT model used here treats periods of sleep as unusually long time-intervals between measurements, relative to the within-day intervals, the carry-over from the last measurement before sleep on the first measurement the next day will be practically zero, ${\varvec{\Phi }}(\Delta t)$ becoming a zero matrix when $\Delta t$ is large enough. As a result, the best prediction for the first observation of the day will be (approximately) equal to the system’s equilibrium, and hence, this lagged relation will essentially not contribute anything to the estimation of the parameters in $\mathbf {A}$. An alternative approach would be treat observations as nested within-days, however, de Haan-Rietdijk et al. (2016) found, in two similar empirical experience sampling datasets, that such an approach did not yield better fitting models than the approach of treating observations as drawn from a single ongoing process.

Ultimately, whether any psychological process is truly evolving continuously over time, is a difficult question to even begin answering, although many researchers have advocated such a viewpoint (e.g., Boker & Nesselroade, 2002). Either way, it is our view that CT models such as considered in this paper could form a more appropriate to the underlying dynamic system than the DT models that are currently in use.

### Future Directions

While the current paper focuses on stationary single-subject models, the approach outlined here could be extended in much the same ways as DT-VAR models have been extended in the psychological literature. For example, CT-VAR models with time-varying parameters could be developed, however, these would suffer from the same limitations as their DT counterparts, such as a need for large sample sizes (Haslbeck et al., 2017). Multilevel CT models that allow for individual differences in means, drift matrices and residual variances can be estimated with existing software packages (Driver & Voelkle, 2018a; Oravecz et al., 2011). In multilevel DT-VAR model applications, there tends to be a primary interest in the individual within-person parameter estimates and/or, for instance, the average of these in different groups (Bringmann et al., 2013; Schuurman, et al., 2016; Asparouhov et al., 2018; Suls et al., 1998; Lodewyckx et al., 2011; Liu et al., 2019). However, psychological network researchers also often construct additional networks based on the inverse covariance matrix of the residuals (sometimes referred to as the “contemporaneous network”) (Epskamp et al., 2018). From a CT perspective however, the residual variances and covariances are also a nonlinear function of the time-interval and the drift matrix (cf. Voelkle et al., 2012; Driver & Voelkle, 2018a), and so exploring their time-interval dependency may also yield valuable insights.

For didactic purposes, we focused mainly on the interpretation of point estimates in the empirical example, ignoring the quantification of uncertainty around these parameters, and broader issues around model selection and inference in general. In practice it is possible to obtain credible or confidence intervals for the drift matrix parameters (as well as $\varvec{\hat{\Phi }}(\Delta t)$ and centrality measures) using posterior sampling in a fully Bayesian approach or by re-sampling from the likelihood using a frequentist approach (as implemented in *ctsem* Driver & Voelkle, 2018a). The *R* package that accompanies this paper (*ctnet*) includes functions which automate this for users.

Finally, the general framework of CT modeling has much more to offer than presented here. Differential equation models are highly flexible, allowing for systems that exhibit more substantively interesting dynamic behaviors, such as the presence of multiple attractors (Strogatz, 2015; Haslbeck & Ryan, in press). Depending on the model, there may be a variety of different effects that a researcher could aim to bring about using interventions, such as shifting the system into a second attractor basin, or changing the attractor landscape to make unhealthy states less likely. To learn about those interventions however, requires more complex models which allow for those qualitative patterns (Haslbeck & Ryan, in press; Haslbeck et al., in press). A number of recent papers have called for an increased focus on theoretical and computational models in psychology (Smaldino, 2017; Navarro, 2020; Guest & Martin, 2020; Borsboom et al., 2020; Robinaugh et al., 2021) with some using differential equations as a framework to build these models (Robinaugh et al., 2019; Haslbeck et al., in press). We welcome these developments, and believe that the ideas outlined in this paper, such as the interpretation of simple continuous-time processes, the notion of pulse and press interventions, and the connections between differential equation models, networks and path models, can serve as a stepping stone between current empirical practice and more substantively interesting dynamic systems models.

### Supplementary Information

Below is the link to the electronic supplementary material.

Supplementary material 1 (pdf 3540 KB)

## Figures and Tables

**Fig. 11:**
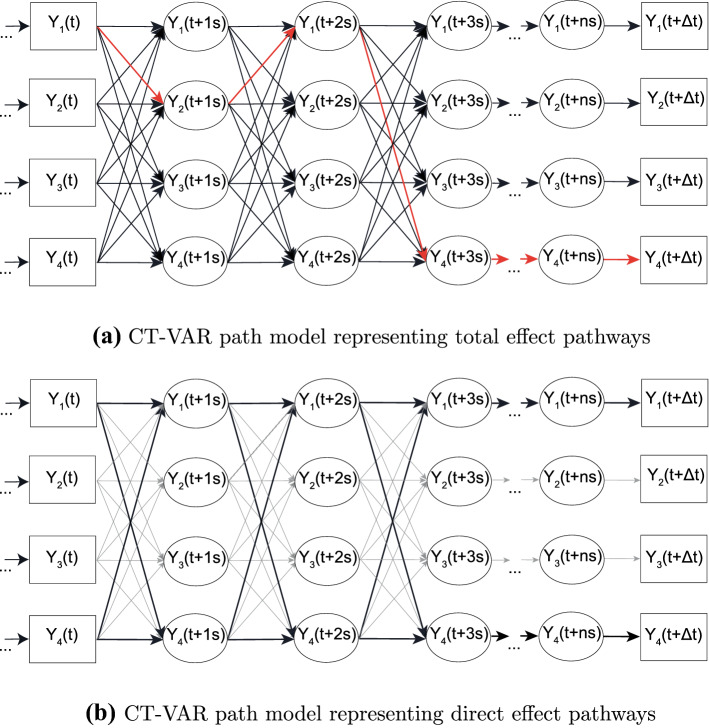
Path-model representation of the four-variable CT-VAR model with full drift matrix ${\varvec{A}}$. In the top panel, the red pathway highlights a path which is included in the difference-method calculation of the indirect effect but omitted from the path-method calculation. The bottom panel shows the pathways which make up the direct effect, with indirect paths (removed from the altered drift matrix ${\varvec{A}}^{(D \left[ -k\right] )}$) shaded in gray (Color figure online).

## Centrality Measures as Summaries of Path-Specific Effects

In this appendix, we show how path-specific effects in DT-VAR models are related to three popular centrality measures calculated from DT-VAR networks. For the measures typically interpreted as quantifying the total and direct influence of a variable (i.e., both Expected Influence measures), this relationship is quite straightforward, while for the popular indirect influence measure Betweenness Centrality, the relationship with path-tracing quantities is much farther removed.

In Table 2, we provide the formula and description of the three centrality measures we consider in the main text. These are expressed in terms of lagged regression parameters $\phi _{ji}$, which represent the lagged effect from process *i* to process *j* (i.e., it is the element on the *j*th row and *i*th column of the matrix $\varvec{\Phi }$). The right-hand column of Table 2 describes how these calculations relate to path-tracing quantities from the SEM literature. Note that the Expected Influence measures were originally developed for undirected networks (Robinaugh et al., 2016), and so, despite the active applications of those measures for directed networks (e.g., Kaiser & Laireiter, 2018) their precise definition for direct networks is left somewhat ambiguous. For instance, the popular packages *qgraph* (Epskamp et al., 2012) and *networktools* (Jones, 2018) differ slightly in how One-Step Expected Influence is calculated, with the former excluding diagonal elements (i.e., auto-regressive effects) as is common for DT-VAR centrality measures, while the latter includes those elements. The definitions we give here to the One-Step and Two-step Expected Influence measures (${\textit{EI}}^{(1)}_i$ and ${\textit{EI}}^{(2)}_i$) omit relationships a variable has with itself either one or two occasions later, respectively. We believe this is in keeping with the spirit of how these measures are defined for undirected networks, and allows us to maintain the standard interpretation of centrality measures as reflecting a type of relationship the target variable shares with all *other* variables in the model.

**Table 2:** Relationship between different network metrics and path-tracing quantities, in the context of a VAR(1) model with *p* variables, regression coefficient matrix $\varvec{\Phi }$, and corresponding dynamical network with weights matrix $\varvec{\Phi }^T$.

Network Measure	Formula	Description
${\textit{EI}}^{(1)}_{i}$ *One-Step* *Expected Influence*	$\sum ^{p}_{j \ne i} \phi _{ji}$	Sum of lag 1 *direct effects* $Y_{i,\tau } \rightarrow Y_{j,\tau + 1}$ $\forall j \ne i$
${\textit{EI}}^{(2)}_{i}$ *Two-Step Expected Influence*	$\sum ^{p}_{j} \phi _{ji} \sum ^p_{k \ne i} \phi _{kj} + {\textit{EI}}_{1i}$	Sum of *total effects* at lag 2 $Y_{i,\tau } \rightarrow Y_{j,\tau + 1} \rightarrow Y_{k,\tau + 2}$ $\forall k \ne i$ plus *direct effects* at lag 1
${\textit{BC}}_{i}$ *Betweenness Centrality*	$M_{jk}(i) =1$ iff $Y_i \in d(jk)$ $\sum \sum _{k \ne j\ne i}^{p} M_{jk}(i)$ where *d*(*jk*) is ${\textit{max}} \{|\phi _{hj}| + .... +|\phi _{kh}| \}$	Counts how often $Y_i$ is a mediator on the shortest *network-path* $Y_{j,\tau } \rightarrow \dots Y_{i,\cdot } \dots \rightarrow Y_{k,\tau + q}$

From Table 2, we can see that ${\textit{EI}}^{(1)}_{i}$, which is typically interpreted as a summary of direct effects, is in fact the sum of lag-one direct effects of $Y_{i,\tau }$ on all other variables at the next occasion (that is, excluding the auto-regressive direct effect of $Y_i$ on itself at the next occasion). The ${\textit{EI}}^{(2)}_{i}$ measure, which is typically interpreted as reflecting the total influence of a variable, comprises two separate parts. The first part is the sum of lag-two total effects, following standard path-tracing rules, and excluding the total effect of a variable on itself two occasions later. The second part is the ${\textit{EI}}^{(1)}_{i}$ measure for that variable. As such, ${\textit{EI}}^{(2)}_{i}$ measure is a mix of total and direct effects at both lags.

Finally, the Betweenness Centrality measure ${\textit{BC}}_{i}$, typically interpreted in terms of indirect effects, is only tenuously related to path-tracing quantities. In SEM approaches, researchers are typically interested in mediators of indirect effects, where the size of an indirect effects is defined by the product of the component pathways (i.e., path-tracing rules). If we have many indirect pathways, and many potential mediators, we may wish to know which specific indirect effect is strongest, and in turn, how often a specific variable acts as a mediator of these strongest indirect effects. It seems that this is how psychological researchers using the ${\textit{BC}}_{i}$ measure typically interpret it (e.g., Bringmann et al., 2013, 2015; David et al., 2018). However, the actual calculation of this measure differs greatly from the mediator-based metric described above. Specifically, instead of identifying the largest indirect effect, Betweenness is based on the identification of the *shortest network-path* between two variables (*d*(*jk*)). The length of this network-path is based on the inverse of the *sum* rather than the *product* of the individual pathways: while large SEM paths are those where multiplying each individual part leads to a large number, we say that short network-paths are those where the sum of each individual part leads to a small number. Similar to standard path-tracing nomenclature, these network-paths can be either direct (e.g., $Y_{j,\tau } \rightarrow Y_{k,\tau + 1} = \phi _{kj}$) or indirect (e.g., $Y_{j,\tau } \rightarrow Y_{i,\tau +1} \rightarrow Y_{k,\tau +2} = \phi _{ij} + \phi _{ki}$) and each path may span a different number of measurement occasions. The Betweenness Centrality of $Y_i$ is found by first calculating all the shortest paths between all pairs of variables, and then counting how often $Y_i$ lies on that shortest path. It is clear then that, despite how this measure is interpreted the relationship of Betweenness Centrality with path-specific effects is much less direct than for the other measures considered above.

## The Matrix Exponential as Path-Tracing

In this appendix, we describe in more detail the relationship between the CT-VAR or Ornstein–Uhlenbeck model, and the notion of path-tracing effects. The CT-VAR model is the integral form of the first-order stochastic differential equation (SDE) model, defined as

14 $$\begin{aligned} \frac{\mathrm{d}{\varvec{Y}}(t)}{\mathrm{d}t} = {\varvec{A}}{\varvec{Y}}(t) + {\varvec{W}}(t) \end{aligned}$$

where ${\varvec{A}}$ is the drift matrix which regresses the derivative on the value of the process at that moment in time, and ${\varvec{W}}(t)$ represents the stochastic innovation part of the system, also referred to as a Wiener process (which is often denoted ${\varvec{G}} \tfrac{\mathrm{d}{\varvec{W}}(t)}{\mathrm{d}t}$, cf. Oud & Jansen, 2000; Voelkle et al., 2012; Voelkle & Oud, 2013). The elements of the drift matrix encode direct dependencies between time-varying processes, with $a_{ij}$ representing the direct effect of $Y_j(t)$ on the rate of change of $Y_i(t)$.

The first derivative $\tfrac{\mathrm{d}{\varvec{Y}}(t)}{\mathrm{d}t}$ is defined as the change in value of ${\varvec{Y}}(t)$ over the time-interval $t + s$, as the value of *s* approaches zero

$$\begin{aligned} \frac{\mathrm{d}{\varvec{Y}}(t)}{\mathrm{d}t} = \lim _{s \rightarrow 0}\frac{{\varvec{Y}}(t+s)-{\varvec{Y}}(t)}{s} \end{aligned}$$

which means that the deterministic part of the first-order differential equation (i.e., ignoring the stochastic innovation part) can be rewritten as

$$\begin{aligned} \lim _{s \rightarrow 0}\frac{{\varvec{Y}}(t+s)-{\varvec{Y}}(t)}{s} = {\varvec{A}}{\varvec{Y}}(t) \end{aligned}$$

Re-arranging, we can come to an expression for the relationship between ${\varvec{Y}}(t)$ and ${\varvec{Y}}(t+s)$, as $s \rightarrow 0$

$$\begin{aligned} {\varvec{Y}}\left( t+\lim _{s \rightarrow 0}s\right)&= \lim _{s \rightarrow 0} s \times ({\varvec{A}}{\varvec{Y}}(t)) + {\varvec{Y}}(t) \\&= \left( {\varvec{I}} + {\varvec{A}}\lim _{s \rightarrow 0}s\right) {\varvec{Y}}(t) \end{aligned}$$

that, is, an expression of the differential equation model as an auto-regressive model of measurements spaced very closely in time. Thus, the auto-regressive and cross-lagged relationships between waves spaced an infinitesimally small time-interval apart (i.e., the moment-to-moment lagged relationships) are given by ${\varvec{I}} + {\varvec{A}}\lim _{s \rightarrow 0}s$. This also shows that the off-diagonal elements, that is, cross-effects, of ${\varvec{A}}$ can be interpreted the same way as cross-lagged effects defined on the moment-to-moment time-interval, since the addition of the identity matrix ${\varvec{I}}$ in the auto-regressive form affects only the diagonal auto-effects.

Now take it that we are interested in finding an expression relating two observed waves of variables ${\varvec{Y}}(t)$ and ${\varvec{Y}}(t+ \Delta t)$. We can think of *s* as a very small fraction of $\Delta t$, that is,

$$\begin{aligned} s = \frac{\Delta t}{n} \end{aligned}$$

such that as $n \rightarrow \infty$ we get $s \rightarrow 0$. This means that we can re-express the relationship between waves spaced an infinitely small time-interval apart as

$$\begin{aligned} {\varvec{Y}}\left( t+\lim _{n \rightarrow \infty } \tfrac{\Delta t}{n}\right) = \left( {\varvec{I}} + {\varvec{A}}\lim _{n \rightarrow \infty }\tfrac{\Delta t}{n}\right) {\varvec{Y}}(t). \end{aligned}$$

Now, if we conceptualize the CT-VAR as a path model, as depicted in Fig. 2 in the main text, then we can find an expression to relate ${\varvec{Y}}(t)$ and ${\varvec{Y}}(t+ \Delta t)$ by a simple application of path-tracing rules (Bollen, 1987). That is, we can trace through the $\lim _{n \rightarrow \infty } n$ latent waves in-between those two occasion, by taking the appropriate power of the moment-to-moment lagged-effects matrix ${\varvec{I}} + \lim _{n \rightarrow \infty } {\varvec{A}}\frac{\Delta t}{n}$. This path-tracing operation gives us

$$\begin{aligned} {\varvec{Y}}(t+\Delta t)&= \lim \nolimits _{n \rightarrow \infty }\left( {\varvec{I}} + \lim _{n \rightarrow \infty } {\varvec{A}}\tfrac{\Delta t}{n}\right) ^n {\varvec{Y}}(t) \\&= \lim \nolimits _{n \rightarrow \infty } \left\{ \left( {\varvec{I}} +\tfrac{1}{n} {\varvec{A}}\Delta t\right) ^n \right\} {\varvec{Y}}(t). \end{aligned}$$

By definition, the first term on the right-hand side is exactly the matrix exponential (cf. Abadir & Magnus, 2005, p. 250)

15 $$\begin{aligned} {\varvec{e}}^{{\varvec{A}}\Delta t} = \lim \nolimits _{n \rightarrow \infty } \left\{ \left( {\varvec{I}} +\tfrac{1}{n} {\varvec{A}}\Delta t\right) ^n \right\} , \end{aligned}$$

giving us

16 $$\begin{aligned} {\varvec{Y}}(t+\Delta t) = {\varvec{e}}^{{\varvec{A}}\Delta t} {\varvec{Y}}(t), \end{aligned}$$

which gives us the deterministic part of the CT-VAR(1) model.

This derivation shows that the CT-VAR model can be seen as a path model, where the lagged relationships are defined as total effects resulting from path-tracing through an $n \rightarrow \infty$ latent waves. Thus, any DT cross-lagged parameter matrix $\varvec{\Phi }(\Delta t) = {\varvec{e}}^{{\varvec{A}}\Delta t}$ should be interpreted as reflecting *total effects* relative to the CT-VAR model.

## Path-Tracing in CT models

In this appendix we describe the calculation of path-specific effects for the CT-VAR model based on path-tracing rules. Both Deboeck and Preacher (2016) and Aalen et al. (2016) describe a method for calculating direct, indirect and total effects in a CT-VAR model, which follow the path-tracing rules laid out by, among others, Bollen (1987). However, these authors only discuss path-tracing with respect to a lower-triangular tri-variate drift matrix, that is, a drift matrix with only three variables and without reciprocal lagged relationships. Here, we generalize these path-tracing definitions to drift matrices of arbitrary structure and number, following path-tracing principles. In large part, we follow the methods described by the original authors, except in the case of the indirect effect, where non-triangular drift matrices must be approached differently than was done in the simpler scenario of a lower-triangular matrix.

To find the path-tracing total effect of $Y_i(t)$ on $Y_j(t+\Delta t)$, which we will here denote ${\textit{TE}}_{ij}(\Delta t)$, we simply take the element in the *j*th row, *i*th column of the matrix exponential of the drift matrix:

17 $$\begin{aligned} {\textit{TE}}_{ij}(\Delta t) = {{\varvec{e}}^{{\varvec{A}} \Delta t}} _{\left[ ji\right] }. \end{aligned}$$

This follows from the interpretation of the matrix exponential term ${\varvec{e}}^{{\varvec{A}}\Delta t}$ as a path-tracing operation, relative to the moment-to-moment auto-regressive effects matrix $({\varvec{I}} + {\varvec{A}}\lim _{n \rightarrow \infty }\tfrac{\Delta t}{n})$ (described in Appendix B). In Fig. 11a, we show a four-variable CT-VAR model with a full ${\varvec{A}}$ matrix in path-model form, with $n \rightarrow \infty$ latent values of the processes in between measurement occasions, spaced at intervals of $s \rightarrow 0$. From this it is clear that tracing a path from, for instance, $Y_1(t)$ to $Y_4(t+\Delta t)$ includes paths through latent values of $Y_1$ and $Y_4$, (e.g., $Y_1(t) \rightarrow Y_1(t+1s ) \rightarrow Y_1(t+ 2s) \rightarrow Y_4(t+ 3s) \rightarrow \dots \rightarrow Y_4(t + \Delta t)$) as well as paths through latent values of $Y_2$ and $Y_3$ ($Y_1(t) \rightarrow Y_2(t+1s) \rightarrow Y_3(t+ 2s) \rightarrow Y_4(t+ 3s) \rightarrow \dots \rightarrow Y_4(t + \Delta t)$). As such, we can interpret this total effect as constituted of all possible pathways linking $Y_1(t)$ and $Y_4(t+\Delta t)$, as is the standard interpretation of a total effect.

In order to find the path-tracing *direct* effect from $Y_i(t)$ to $Y_j(t+\Delta t)$ relative to some mediator variable(s) $Y_k$, Deboeck and Preacher (2016) state that the drift matrix should first be altered so that the parameters which make up the indirect pathways are omitted. We can alter the drift matrix to achieve this by setting the *k*th row and column elements of ${\varvec{A}}$ to zero, yielding a drift matrix containing only direct relationships between $Y_i$ and $Y_j$, which we will denote ${\varvec{A}}^{(D \left[ -k\right] )}$. The path-tracing direct effect is then found by applying the matrix exponential function to the altered drift matrix.

18 $$\begin{aligned} {\textit{DE}}_{ij \cdot k}(\Delta t) = {{\varvec{e}}^{{\varvec{A}}^{(D[-k])} \Delta t}} _{\left[ ji\right] }. \end{aligned}$$

For example, for a four-variable system, to define the path-tracing direct effect of $Y_1(t)$ to $Y_4(t+\Delta t)$ relative to the mediators $Y_2$ and $Y_3$ we would need to alter the drift matrix as follows

This altered drift matrix defines a new path model, absent of any lagged relationships linking $Y_1$ to $Y_4$ through the mediators $Y_2$ and $Y_3$. This is displayed in Fig. 11b. Applying the matrix exponential function to this new drift matrix, it is clear that we only trace through direct pathways linking $Y_1(t)$ to $Y_4(t+\Delta t)$ (e.g., $Y_1(t) \rightarrow Y_1(t+1s) \rightarrow Y_1(t+ 2s) \rightarrow Y_4(t+ 3s) \rightarrow \dots \rightarrow Y_4(t + \Delta t)$). This process is exactly equivalent to how Bollen (1987) describes the calculation of a direct effect using matrix algebra.

To calculate the indirect effect for a lower-triangular drift matrix, both Deboeck and Preacher (2016) and Aalen et al. (2016) describe an operation by which the direct links are omitted from the drift matrix (in the four-variable example, this would be $a_{14}$ and $a_{41}$) before applying the matrix exponential term. We will refer to this as the *trace method* of calculating an indirect effect. Alternatively, following path-tracing rules in linear models, we could define the indirect effect as the difference between the total and direct effect, which we will refer to as the *difference method*. For a lower-triangular drift matrix, both methods yield the same indirect effect (Deboeck & Preacher, 2016).

However, for non-triangular drift matrices, these definitions will not be equivalent. The reason again follows simple path-tracing rules. The difference method in this scenario quantifies all paths from $Y_i(t)$ to $Y_j(t + \Delta t)$ that pass through some latent value of $Y_k$. In contrast, the trace method quantifies fewer paths, that is, all paths that pass through $Y_k$, but *do not* pass along any direct paths linking $Y_i(t)$ to $Y_j(t+s)$. In Fig. 11a, we have highlighted in red a pathway which is included as part of the difference-method indirect effect, but which is not included in the trace-method indirect effect.

In order to maintain the property that the total and direct effects sum to one another, and to allow an easier link to intervention-based definitions of indirect effects in Sect. 3.2 of the main text, we recommend the use of the difference method of calculating indirect effects. As such, we define the path-based indirect effect as

19 $$\begin{aligned} {\textit{IEP}}_{ij}(\Delta t) = {{\varvec{e}}^{{\varvec{A}} \Delta t}} _{\left[ ji\right] } - {{\varvec{e}}^{{\varvec{A}}^{(D[-k])} \Delta t}} _{\left[ ji\right] }, \end{aligned}$$

which is equivalent to the difference between the path-tracing total effect and the path-tracing direct effect described above.

## Interventions and Path-Tracing in CT Models

In this appendix, we prove the equivalence between the intervention-based definitions of total, direct and indirect effects, and the path-tracing definitions of these quantities (described in Appendix C). In contrast to the typical approach taken in the causal inference literature, in which strict identifiability assumptions are explicated in order to identify the effects of these interventions from the observational distribution (cf. Pearl, 2009; Dawid, 2010) we here take a highly simplified approach, for instance assuming throughout that intervening on the system does not change how variables relate to one another (known as the modularity assumption), that the system is fully observed (sufficiency) and that the CT-VAR model correctly describes the dynamics of the underlying system. As such, the equivalence between intervention-based and path-tracing definitions given below can be considered to hold under highly idealized conditions.

### Total Effect

We define the total effect of $Y_i(t)$ on $Y_j(t + \Delta t)$ as the expected change in value of $Y_j(t+\Delta t)$ given pulse intervention to set the value of $Y_i(t)$ from a constant, $y_i^*$ to a new value $y_i$. We denote such a variable-setting operation using the *do* operator (Pearl, 2009), and so can express this total effect as

20

By assumption, we substitute the expected value of $Y_j(t+\Delta t)$ following an intervention $do(Y_{i}(t) = y_i)$ with the expected value given we observe $Y_i(t) = y_i$. This yields the expression

Now we plug in the CT-VAR model for those expected values. Take it that ${\varvec{Y}}(t)$ represents a column vector of variable values with *i*th element $Y_{i}(t)=y_i$. Using this, we can express the first expected value as

that is, the *j*th element of the column vector obtained by multiplying the square matrix ${\varvec{e}}^{{\varvec{A}}\Delta t}$ with the column vector ${\varvec{Y}}(t)$. To obtain the second expectation, we take it that $\varvec{Y^*}(t)$ represents a column vector of variable values with *i*th element $Y_{i}(t)=y_i^*$ but which is otherwise identical to ${\varvec{Y}}(t)$.

Taking the difference between these two expected values, we obtain

$$\begin{aligned} {\textit{TE}}_{ij}(\Delta t)&=\{{{\varvec{e}}^{{\varvec{A}}\Delta t} {\varvec{Y}}(t)}\}_{[j]} - \{{{\varvec{e}}^{{\varvec{A}}\Delta t} \varvec{Y^*}(t)}\}_{[j]} \\&= {{\varvec{e}}^{{\varvec{A}}\Delta t} \cdot \big ({\varvec{Y}}(t) - \varvec{Y^*}(t) \big )}_{[j]}. \end{aligned}$$

Since the vectors differ only with respect to their *i*th element, we obtain

21 $$\begin{aligned} {\textit{TE}}_{ij}(\Delta t) = {{\varvec{e}}^{{\varvec{A}}\Delta t}}_{\left[ ji\right] } \times (y_i - y_i^*) \end{aligned}$$

where ${{\varvec{e}}^{{\varvec{A}}\Delta t}}_{\left[ ji\right] }$ is the element in the *j*th row and *i*th column of the matrix ${\varvec{e}}^{{\varvec{A}}\Delta t}$. If we define the intervention as increasing the value of $Y_i(t)$ by one unit ($y_i - y_i^* = 1$), this yields an expression exactly equivalent to the path-tracing definition of a total effect given in Appendix C.

### Direct Effect

We define the direct effect of $Y_i(t)$ on $Y_j(t + \Delta t)$ as the expected change in value of $Y_j(t+\Delta t)$ given an acute intervention to set the value of $Y_i(t)$ from $y_i^*$ to a new value $y_i$, while also intervening to keep the value of the mediator(s) $Y_k$ fixed to a constant $y_k$ at every moment in time in that interval. We denote this latter press intervention using the *do* operator over an interval of time as $do(\overline{Y_k(t+\Delta t)}=y_k$, and so express the direct effect as

22

for some mediator(s) $k \in p$. Intuitively, if we want to block the indirect effect that acts through a mediator, we would need to ensure that either the mediator does not react to changes in the cause variable, or that it does not transmit information to the effect variable, or both. If we wish to achieve this by intervening on a variable, it is straightforward to see that we must do so by intervening to set the value of the mediator to a constant at every point in time between *t* and $t+\Delta t$.

As with the total effect derived above, the next step consists of plugging in the CT-VAR model for the expected values in this expression. However, note that due to the need to define the press intervention on the mediator $do(\overline{Y_k(t+\Delta t)}=y_k$ this proof is a little more involved than that of the total effect above. To derive an expression for the direct effect, we first begin with the expression for the expected value of ${\varvec{Y}}(t+\Delta t)$ given pulse intervention on the cause variable $Y_i(t)$, that is,

one of the components of the *total effect* given above. Recall from the derivation in Appendix B that we can write the CT-VAR model as describing lagged relationships over an infinitesimally small time-interval $\lim _{n \rightarrow \infty } \tfrac{\Delta t}{n}$, hereby referred to as the moment-to-moment relationship. This gives us

From now, we will treat this expression as defining a moment-to-moment path model, as depicted in Fig. 2 in the main text, and with a slight abuse of notation we will substitute $\lim _{n \rightarrow \infty } \tfrac{\Delta t}{n}$ for *s*, which we will define as a “moment” in time. We can express the expected value two “moments” after *t* as

where the second and third line follow by substituting in the expression for  given above.

Now, to define the direct effect we need to express the expected value of ${\varvec{Y}}(t+2s)$ given that we have intervened to set the current value of the mediator ($Y_k(t+2s)$), the value of the mediator one “moment” previously ($Y_k(t+s)$), *and* the initial value of the mediator $Y_k(t)$ to some constant value. In order to derive such an expression, we introduce two simplifications here. First, since we are focusing on a linear model, and we are interested in the difference between two expected values in which in both cases the mediator $Y_k$ is set to the same value $y_k$, the specific value we choose for $y_k$ is irrelevant. For ease of notation, we will therefore consider only an intervention by which $y_k$ is equal to zero (i.e., the equilibrium position of $Y_k$). Second, to aid in our derivation, we will express the *do* operator in matrix algebraic terms. That is, we will represent the operation $do(Y_k(t) = 0)$ using a transformation matrix ${\varvec{D}}_{[-k]}$, a $p \times p$ matrix with zeros as off-diagonal elements, a zero on the *k*th diagonal element, and ones as the other diagonal elements. For instance, a $3 \times 3$ matrix ${\varvec{D}}_{[-2]}$ would be given as

$$\begin{aligned} \varvec{D_{[-2]}} = \begin{bmatrix} 1 &{} 0 &{} 0 \\ 0 &{} 0 &{} 0 \\ 0 &{} 0 &{} 1 \\ \end{bmatrix}. \end{aligned}$$

Pre-multiplying a column vector by the matrix ${\varvec{D}}_{[-k]}$ reproduces the original column vector but with a zero as the *k*th element. That means that ${\varvec{D}}_{[-k]}{\varvec{Y}}(t)$ denotes the acute intervention $do(Y_k(t) = 0)$. Again, for ease of notation we will drop the $[-k]$ notation and leave it implied, that is, in the proof below, ${\varvec{D}} = {\varvec{D}}_{[-k]}$ unless otherwise specified.

Using this matrix representation of the *do* operator, we can express the expected value of ${\varvec{Y}}(t+2s)$ given that we have intervened to set the current value of the mediator ($do(Y_k(t+2s) =0)$), the value of the mediator one “moment” previously ($do(Y_k(t+s)=0)$), *and* the initial value of the mediator ($do(Y_k(t)=0)$) to zero. Subsequently, since we repeat this acute intervention at every “moment” in time in an interval, we can describe it as a press intervention $do(\overline{Y_k(t+s)}=0)$ that is, an intervention that is present for all possible time points in an interval. The expected value of ${\varvec{Y}}(t+2s)$ given this press intervention can be written as

Now, using the same substitutions as described in Appendix B, we can express the expected value an arbitrary time-interval $\Delta t$ later, given that we intervene to set $Y_k$ to zero at each of the $\lim _{n \rightarrow \infty }$ time points in that interval. This is given by

Noting that ${\varvec{D}}$ is an idempotent matrix, and that ${\varvec{D}}{\varvec{I}}{\varvec{D}} = {\varvec{D}}{\varvec{I}} = {\varvec{I}}{\varvec{D}}$, we can simplify this expression to

which, by the definition of the matrix exponential function simplifies to

23

where ${\varvec{D}} {\varvec{Y}}(t)$ ensures that the initial value of $Y_k(t)$ is set to zero.

Pre- and post-multiplying ${\varvec{A}}$ by $\varvec{D_{[-k]}}$ has the effect of setting the *k*th row and column of ${\varvec{A}}$ to zero. Hence, the expression ${\varvec{e}}^{\varvec{DAD}\Delta t}$ is exactly equivalent to the path-tracing definition of the direct effect given in Appendix C, that is, $\varvec{DAD}={\varvec{A}}^{(D[-k])}$. This implies that by plugging the above expression in for the expected values in the direct effect definition, we obtain

24

which shows that the effect on $Y_j(t+\Delta t)$ of an acute intervention to change $Y_i(t)$ combined with a press intervention to keep the mediator $Y_k$ fixed is identical to the path-tracing direct effect.

It follows from the equivalence between path-tracing and intervention-based direct and total effects that the indirect effect, defined as a contrast between those two, can be calculated by taking the different in path-tracing definitions of each component effect, described in Appendix C.

### General Pulse Interventions

Both from a conceptual standpoint, and from the derivation of the direct effect above, it is clear that a press intervention destroys the paths from other variables to the intervened-on variable. This is achieved by pre-multiplying the drift matrix by the transformation matrix (${{\varvec{D}}}{{\varvec{A}}}$), which denotes that, if a variable is forced to remain constant, it’s rate of change is now independent of the other variables in the model. For the direct effect we consider press interventions which set $Y_k$ to its equilibrium value of zero: if a variable is forced to stay at equilibrium it will not exert any direct influence on the other variables in the system, and so, we can post-multiply by the transformation matrix, yielding $\varvec{DAD}$ in Eq. (23).

However, if the press variable is set to a non-equilibrium value, say *c*, then that variable does exert an influence on all others in the system. To understand the effect of such a press intervention, we first construct the transformation matrix ${\varvec{D}}_{[-k]}$, and then check the eigenvalues of the resulting modified drift matrix ${{\varvec{D}}}{{\varvec{A}}}$. If the real parts of the eigenvalues of ${{\varvec{D}}}{{\varvec{A}}}$ are positive, then this press intervention has created an unstable system. If the real parts of the eigenvalues of ${{\varvec{D}}}{{\varvec{A}}}$ are negative, then the intervened-on system is still stable. In the latter case, we can simulate the effects of such an intervention using the expression

where $Y_k(t) = c$. Plugging in a sufficiently large value of $\Delta t$ will yield the new equilibrium positions of the intervened-on system (though what precisely that value is depends on the system at hand). A function to compute the effects of the press intervention is given in the online supplementary materials and accompanying *R* package, and an example of such a press intervention is shown in the main text in Fig. 5b.

## Empirical Example Parameter Estimates

The parameter estimates for the CT and DT models estimated on empirical data, as discussed in Section 4, are presented in Tables 3 and 4 respectively. Code to reproduce these analyses is available in Online Supplementary Materials 2.

**Table 3:** Estimated drift matrix $\varvec{\hat{A}}$ parameter estimates from the empirical example.

	SD	Ti	Ir	Rl
SD	$-$0.984 ($-$1.322, $-$0.710)	0.036 ($-$0.124, 0.192)	$-$0.675 ($-$1.356, 0.018)	0.791 (0.123, 1.503)
Ti	$-$0.002 ($-$0.222, 0.229)	$-$0.782 ($-$0.911, $-$0.649)	0.294 ($-$0.203, 0.807)	$-$0.105 ($-$0.625, 0.382)
Ir	$-$0.317 ($-$0.755, 0.082)	0.002 ($-$0.225, 0.226)	$-$2.222 ($-$3.379, $-$1.309)	1.343 (0.338, 2.350)
Rl	0.444 (0.160, 0.761)	$-$0.176 ($-$0.327, $-$0.019)	1.007 (0.304, 1.722)	$-$1.605 ($-$2.438, $-$0.994)

Point estimates are given with lower and upper 95% confidence interval in ellipses.

**Table 4:** Estimated DT-VAR lagged parameter matrix $\varvec{\hat{\Phi }}$ from the empirical example.

	SD	Ti	Ir	Rl
SD	0.341 (0.283, 0.400)	$-$0.019 ($-$0.071, 0.033)	$-$0.022 ($-$0.085, 0.046)	0.092 (0.025, 0.166)
Ti	$-$0.022 ($-$0.085, 0.04)	0.298 (0.245, 0.347)	0.064 (0.000, 0.132)	0.021 ($-$0.046, 0.093)
Ir	0.067 (0.007, 0.130)	$-$0.068 ($-$0.119, $-$0.010)	0.156 (0.088, 0.229)	0.124 (0.047, 0.200)
Rl	0.127 (0.064, 0.184)	$-$0.078 ($-$0.128, $-$0.026)	0.098 (0.034, 0.165)	0.264 (0.195, 0.339)

Point estimates are given with lower and upper 95% confidence interval in ellipses.

## Footnotes

1 In order for a process to be stationary, the $\varvec{\Phi }$ matrix eigenvalues must lie within the unit circle (Hamilton, 1994).

2 This is also sometimes referred to as the *lag* problem, both by Gollob and Reichardt (1987) and other authors (e.g., Hamaker & Wichers, 2017). However, the *lag* of a model is often used to refer to the order of a lagged regression model: lag-one relations are between current variables and variables at the exactly preceding measurement occasion, and lag-two relations between current variables and variables two measurement occasions previously, etc.

3 While the forward operation from shorter to longer time-interval matrix is always possible, one can only uniquely “solve” for the shorter time-interval relationships when the longer time-interval matrix has only real-valued eigenvalues. If the matrix has complex eigenvalues (i.e., oscillating behavior), we run into the so-called aliasing problem—we cannot distinguish between structures with higher-frequency oscillations (for details, see Bergstrom, 1984; Hamerle et al., 1991).

4 In fact, taking the power of a matrix of direct effects, as in Eq. (2), is suggested by Bollen (1987) as a method of calculating total effects in the SEM literature, an equivalence which can be seen immediately from the derivation of $\phi _{41}(1)$ in terms of $\varvec{\Phi }(\frac{1}{2})$ parameters given above and by inspecting the path-model representations in Fig. 1a, b.

5 More complex CT models are required to produce patterns of DT negative auto-regression (Fisher, 2001).

6 https://github.com/ryanoisin/ctnet/.

7 In the time-series literature, the idea of setting a time-varying process to a particular value at a particular point in time is referred to as applying an *impulse* to a process, and tools such as impulse response functions are often used to visualize the effect of this impulse over time (Hamilton, 1994).

8 Here, since we are dealing with a single-subject dynamic process, the expectation is defined with respect to the stochastic input, that is, the normally distributed noise process, as is standard in time-series approaches (Hamilton, 1994). We can interpret this as an expectation for an individual over a population of time points *t*. This is analogous to a causal effect for that individual for an unspecified point in time *t*, assuming that the intervention does not change how the variables in the system relate to one another.

9 For instance, to determine the direct effect of Anxiety on Physical Discomfort, we need to keep Stress and Self-Consciousness at a fixed value (e.g., zero); but to determine the direct of Anxiety on Stress, we need to keep Physical Discomfort and Self-Consciousness at a fixed value. Thus, each component direct effect requires different variables to be kept fixed while others can vary.

10 Available from https://osf.io/c6xt4.
