# Supplementary material for: Time to Intervene: A Continuous-Time Approach to Network Analysis and Centrality
Source: Psychometrika. 2021 Jun 24;87(1):214–52. doi: 10.1007/s11336-021-09767-0 (PMC9021117; doi:10.1007/s11336-021-09767-0)
Supplement: Supplementary file 1 — Supplementary material 1 (pdf 3540 KB) [file 11336_2021_9767_MOESM1_ESM.pdf]

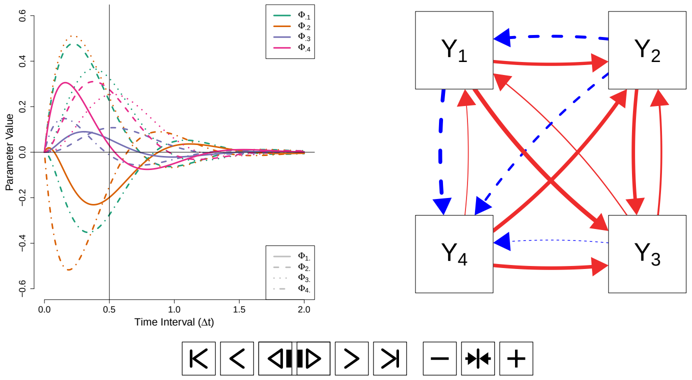
Φ.1
Φ.2
Φ.3
Φ.4
Φ1.
Φ2.
Φ3.
Φ4.
Parameter Value
0.6
0.4
0.2
0.0
−0.2
−0.4
−0.6
0.0
0.5
1.0
1.5
2.0
Time Interval (Δt)
Y1
Y2
Y4
Y3
